# Dynamic changes and interaction between different aroma types during low‐temperature roasting of bud green tea

**DOI:** 10.1002/fsn3.4249

**Published:** 2024-06-17

**Authors:** Cong‐Ming Wang, Xiao‐Qin Tan, Xiao Du, Jia‐Jing Hu, Xin‐Yi Li, Li‐Shu Yan, Xiang Zhang, Cong‐Ning Nie, Liu‐Yi Chen, Feng Du, Yue‐Ling Zhao, Jin‐Lin Bian, Pin‐Wu Li

**Affiliations:** ^1^ Sichuan Agricultural University Chengdu China; ^2^ YiBin Vocational and Technical College Yibin China; ^3^ Tea Refining and Innovation Key Laboratory of Sichuan Province Chengdu China; ^4^ Sichuan Academy of Agricultural Sciences Chengdu China

**Keywords:** aroma perception, bud green tea, interaction, low‐temperature roasting, volatile compounds

## Abstract

As an important process for enhancing aroma of Wuyi rock tea, roasting has gradually been applied to the processing of bud green tea (BGT). However, there is a lack of comprehensive research on the impact of roasting on BGT aroma. This research provides a detailed analysis of the changes in aroma perception and compounds during the low‐temperature roasting process (105°C, 90 minutes) of BGT. First, the changes in aroma perception were studied using quantitative descriptive analysis (QDA). The aroma perception of BGT transformed from fresh to chestnut like. Next, headspace solid‐phase microextraction–gas chromatography–mass spectrometry (HS‐SPME‐GC–MS) analysis was conducted to characterize the volatile compounds during roasting. A total of 313 volatile compounds were identified, among which 72 showed significant differences. Compounds such as linalool, benzyl alcohol, ionone, and ethyl salicylate (floral aroma) and 2,6‐dimethylpyrazine, 2,3‐dimethylpyrazine, 2‐acetylpyrrole, and 3‐furfural (caramel‐like aroma) were confirmed to be involved in the formation of the chestnut‐like aroma during roasting process. In addition, representative aroma compounds with different characteristics were selected based on monomolecular olfactory results to simulate sensory‐level aroma interactions. The results showed that fresh and tender aromas exhibited mutual enhancement at low concentrations, while sweet and chestnut‐like aromas showed mutual enhancement at all concentrations.

## Graphical abstract

The dynamic changes in aroma compounds and aroma sensory during bud green tea (BGT) roasting process were determined. The interaction between aroma sensory in BGT was determined.

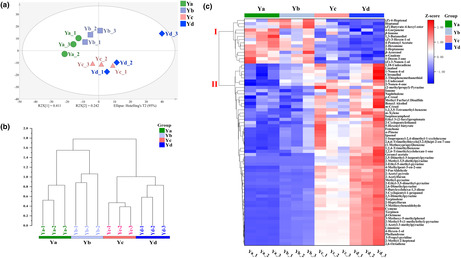

## 1 INTRODUCTION

As a subcategory of green tea, bud green tea (BGT) is a high‐end green tea made entirely from tea plant buds, and its most representative products are Zhu‐ye‐qing and Jintanqueshe tea (Zhang et al., 2020). BGT is mostly known for its neat appearance, emerald green color, and refreshing taste, which attract a large number of consumers. Volatiles in teas are only present in minimum quantities of 0.01% of total dry weight (Yang, 2013), but the aroma still affects the acceptance of a tea before it is tasted (Cai et al., 2016; Cheng et al., 2008; He et al., 2020; Inoue et al., 2016). Therefore, how to improve the aroma quality while ensuring the original characteristics of BGT has become an important direction for its innovative development.

The roasting process has been proven to be an important technique for enhancing the aroma quality of Wuyi Rock tea (Guo, Ho, Schwab, & Wan, 2021; Guo, Ho, Wan, et al., 2021; Liu et al., 2022; Zhan et al., 2023). The impact of roasting on the aroma of Wuyi Rock tea mainly includes high temperature and long time roasting would induce intensive chemical reactions, such as Maillard reaction, caramelization, and degradation of carotenoids, lipids, and glycosides. It was found that full fire roasting can enhance roasted, floral, and woody odors of WRT. Indeed, a substantial number of aldehydes, ketones, and heterocyclic compounds, imparting woody, floral, and nutty scent, were formed during roasting (Liu et al., 2022). Guo et al. explored that 3,5‐dimethyl‐2‐ethylpyrazine was responsible for the roasted attribute of Shuixian WRT after roasting (Guo, Ho, Wan, et al., 2021). Liu et al. analyzed that α‐ionone could be a key characteristic marker for the roasting process (Liu et al., 2022).

Due to the enhancing effect of roasting on the aroma of tea, some tea companies have incorporated roasting techniques into the production process of BGT in order to improve its aroma quality. Unlike the drying process in BGT processing, roasting involves further long‐time high‐temperature treatment of the dried tea leaves. The main purpose of roasting is to enhance the aroma quality of the tea while drying of BGT aims to reduce the moisture content of the tea buds for better transportation and storage. However, due to the significant quality changes in BGT after drying during high‐temperature roasting, it is extremely difficult to control the stability of its quality. Additionally, there is currently a lack of comprehensive research on the impact of roasting on the aroma of BGT. Therefore, the application of roasting techniques for aroma improvement in BGT is still in the preliminary stage, primarily relying on empirical knowledge.

Generally, the roasting process applied to Wuyi Rock tea can be classified into low‐temperature roasting (100–110°C), medium‐temperature roasting (110–130°C), and high‐temperature roasting (130–150°C) (Guo, Ho, Schwab, & Wan, 2021). The preliminary experimental results indicate that medium‐ and high‐temperature roasting greatly alters the color of BGT, resulting in a loss of its characteristic green color and a decrease in its commercial value. Additionally, under medium‐ and high‐temperature roasting, the aroma of BGT undergoes drastic changes and easily develops a burnt smell. On the other hand, low‐temperature roasting allows for a gradual, slow, and controllable transformation of the basic aroma characteristics of BGT, with minimal color change. Therefore, it may be considered that applying low‐temperature roasting to improve the aroma of BGT is currently the most feasible approach.

Therefore, the present study selected low‐temperature roasting as the main approach to improve the aroma of BGT, and explored the dynamic changes in the aroma of BGT during low‐temperature roasting. The main research approach and objective of this study were as follows: Firstly, the sensory changes in the aroma and the changes in volatile compounds of BGT during low‐temperature roasting were determined by using quantitative descriptive analysis (QDA) and headspace solid‐phase microextraction/gas chromatography–mass spectrometry (HS‐SPME/GC–MS). Subsequently, statistical analysis methods such as variance analysis and correlation analysis were employed to determine the correlation between the volatile compounds and sensory perception, as well as identify the key volatile compounds contributing to the variations in sensory perception during the roasting process of BGT. Furthermore, through in vitro‐simulated interactive experiments, the mutual influence of representative compounds with different aroma types on the sensory level was studied. This research will help us comprehensively understand the aroma changes in BGT during the roasting process and provide useful information for practical manufacturing, aiming to move away from empirical knowledge and ensure a high and reproducible quality of BGT.

## 2 MATERIALS AND METHODS

### 2.1 Tea sample

Sample preparation: buds from Fuding Dabai tea plant were used to obtain samples. Sample preparation was conducted based on the method proposed by our previous research (Wang, Du, et al., 2022), which included the steps of spreading (room temperature, 9 h), fixing (280°C, 120 S), shaping (110°C, 35 min), and drying (100°C, 20 min). Detailed parameters for sample preparation are reported in Table S1 and Figure S1. After drying, samples were sealed and stored at −4°C in a refrigerator for further processing.

Sample roasting processing: For roasting, dried samples were roasted at 105°C for 90 minutes; throughout this period, samples were collected every 5 min with a total of 19 samples, which were labeled from 1 to 19. The roasting of the samples was carried out in an HC‐H0802 rotary tea roasting machine (Hebei Xincai Technology Development Co., Ltd., China). In order to minimize the time required for each sampling, the tea leaf samples were predivided into 19 portions before roasting. After roasting, the tea samples are stored in a −4°C refrigerator. Half of these samples are used for sensory evaluation of tea, while the other half is used for HS‐SPME/GC–MS determination of tea. In order to reduce the impact of storage on the aroma of tea, sensory evaluation of tea aroma is conducted immediately, and tea samples used for HS‐SPME/GC–MS determination are transferred to a −80°C refrigerator for storage.

Sample screening: The triangle test method was used to screen samples with significant sensory differences. Ten skilled/experienced sensory evaluators (six men and four women aged 23–29—recruited at the Food College of Sichuan Agricultural University, China.) were selected to screen samples in sequence, and the triangle test method was employed to screen samples with significant sensory differences compared with the previous sample. In triangle test, a significance test (ɑ = 0.05) should be conducted based on the correct number of evaluators to determine whether there is a difference; when there is a significant difference between two samples, then the sample was considered to significantly differ from the previous sample. Finally, four samples with the original labels 1 (initial sample), 4 (significant sensory difference with sample “1”), 11 (significant sensory difference with sample “4”), and 16 (significant sensory difference with sample “11”) were screened and relabeled as Ya (0 min), Yb (15 min), Yc (50 min), and Yd (75 min). The screening was conducted at least three times independently.

### 2.2 Reagents

The following chemicals were purchased from Sigma‐Aldrich (Shanghai, China), with a purity of ≥99%, including ethyl decanoate for the determination of volatile compounds in tea; (Z)‐3‐hexen‐1‐ol, (2E,4E)‐2,4‐decanedienal, 1‐octanol, 2‐ethyl‐3,5‐dimethylpyrazine, 2‐hexenal, heptaldehyde, nerolidol, 2,6‐dimethylpyrazine, (Z)‐3‐hexenyl acetate, dimethyl sulfide, furaneol, and 2‐acetyl pyrrole for in vitro‐simulated interaction experiments.

### 2.3 Sensory analysis

Quantitative descriptive analysis (QDA) was used for sensory evaluation of tea, and the QDA method referred to Mao (Mao et al., 2018) and Wang (Wang, Dai, et al., 2022; Wang, Du, et al., 2022) with appropriate modifications. In brief, 10.0 g of tea sample was placed into a 250‐mL triangular flask, heated for 10 min in a water bath at 50 ± 1°C to ensure that the aroma diffused into the bottle saturated the space, and then immediately assessed by sniffing in a sensory panel room at 25 ± 1°C. The evaluators were asked to judge the samples based on the aroma attributes of fresh (reference standard aroma component is (Z)‐3‐hexenol), tender (nonanal), chestnut like (3‐methylbutyraldehyde), sweet (phenylethanol), and flower (linalool) on a scale from 0 to 3 (0 odorless, 3 strongest). The detailed description and explanation of flavor are summarized in Table S2. All experiments were repeated three times. The prepared samples and hydrolyzed infusions were scored by a panel of 10 trained evaluators (6 men and 4 women aged 23–29) recruited at the Food College of Sichuan Agricultural University, China. The sensory evaluation room enabled conducting the analysis independently, with each evaluator separated by a board 2 meters apart. The basic characteristics of the sensory room meet the relevant requirements of the Chinese national standard “GB/T 18797‐2012 General requirement of the sensory test room.” The area of the sensor room is 40 m^2^, with white walls and ceilings, gray white floors, and no odor indoors. The illumination of the evaluation desk is 1500 lx, and the indoor temperature is maintained at around 25°C, with a relative humidity of less than 70%. During sensory analysis, evaluators were requested not to interact with each other.

### 2.4 Determination of volatile compounds

A headspace solid‐phase microextraction–gas chromatography–mass spectrometry (HS‐SPME‐GC–MS) approach was applied to quantify volatile compounds in tea samples.

For HS‐SPME, tea samples were harvested, weighed, immediately frozen in liquid nitrogen, and stored at −80°C until needed. Then, samples were ground to powder in liquid nitrogen, and 1 g of the obtained powder was transferred immediately to a 20 mL headspace vial (Agilent, Palo Alto, CA, USA) containing a saturated NaCl solution and 10 μL of ethyl decanoate internal standard solution. Each vial was sealed with crimp‐top caps and TFE‐silicone headspace septa (Agilent), and then placed at 60°C for 5 min and analyzed using a 120 μm DVB/CWR/PDMS fiber (Agilent) for 15 min at 60°C. The length of SPME fibers and the depth of fibers entering the top space are both 2 cm.

After sampling, volatile compounds desorption from the fiber was carried out in the injection port of the GC apparatus at 250°C for 5 min in splitless mode. The identification and quantification of volatile compounds were carried out in an Agilent 8890 GC and 7000 D mass spectrometer (Agilent) equipped with a 30 m × 0.25 mm × 0.25 μm DB‐5MS (5% phenyl‐polymethylsiloxane) capillary column. Helium was used as the carrier gas at a linear velocity of 1.2 mL/min. The injector temperature was 250°C and the detector temperature at 280°C. The oven temperature was kept at 40°C for 3.5 min, then increased at a rate of 10°C/min to 100°C, then at a rate of 7°C/min to 180°C, then at a rate of 25°C/min to 280°C, and hold for 5 min. Mass spectra were recorded in electron impact (EI) ionization mode at 70 eV. The quadrupole mass detector, ion source, and transfer line temperatures were set, respectively, at 150°C, 230°C, and 280°C. MS in ion monitoring (SIM) mode was used for the identification and quantification of volatile compounds. The identification of the compounds was achieved by comparing the retention indices and mass spectra. A compound was considered to be identified if the similarity between mass spectrometric in formation of each chromatographic peak and the National Institute of Standards and Technology (NIST 11) mass spectra library was at least 75%, and the difference between experimental retention indices and the scientific literature retention indices did not exceed 30 units.

### 2.5 Simulation experiment of aroma interactions

Aroma interaction stimulation experiments were conducted based on the method reported by Nie (Nie et al., 2020). with a few modifications: solutions of various aroma monomers and aroma types were prepared based on different threshold (recognition threshold) multiples (0, 0.5, 1, 5, 10, and 50), and prepared solutions were then mixed at a ratio of 1:1 (*v/v*). The mixtures were stored in a closed container and heated at a constant temperature (50°C) for 10 min. Subsequently, the five evaluators evaluated the aroma intensities of the mixed solution using a score of 0–3 (0 odorless, 3 strongest). Moreover, the concentrations at which the aroma type shifting or masking occurred were recorded. All experiments were repeated three times independently.

The screening method for representative aroma monomers corresponding to different aroma types: based on odor activity value (OAV) results (Table S3) and literature screening results, 10 possible representative aroma monomers were selected for each aroma type. Then, five trained evaluators smelled the aroma of these monomers and provided one to three descriptive words, Finally, three aroma monomers corresponding to each aroma type were determined based on the frequency of corresponding descriptive words (Table 1).

**TABLE 1:** Screening results of volatile compounds corresponding to different aroma types.

No.	Aroma type	Aroma compound	CAS
1	Fresh	(*Z*)‐3‐Hexen‐1‐ol (Leaf alcohol)	928‐96‐1
		2‐Hexenal	6728‐26‐3
		(*Z*)‐3‐Hexenyl acetate (Leaf acetate)	3681‐71‐8
2	Tender	(2*E*,4*E*)‐2,4‐Decanedienal	25,152‐84‐5
		Heptaldehyde	111‐71‐7
		Dimethyl sulfide	75‐18‐3
3	Sweet	1‐Octanol	111‐87‐5
		Nerolidol	7212‐44‐4
		Furaneol	3658‐77‐3
4	Chestnut like	2‐Ethyl‐3,5‐dimethylpyrazine	55,031‐15‐7
		2,6‐Dimethylpyrazine	108‐50‐9
		2‐Acetyl pyrrole	1072‐83‐9

### 2.6 Statistical analysis

Principal component analysis (PCA) and hierarchical cluster analysis (HCA) were used to qualitatively analyze the differences in aroma sensory and aroma composition among different tea samples. One‐way analysis of variance (ANOVA) was used to screen for significant differences in volatile compounds (*p* < .05). Partial least squares regression (PLSR) was used to analyze the correlation between volatile compounds and aroma sensory perception, with the independent variable “x” as 72 significantly different volatile compounds and the dependent variable “y” as 5 aroma sensory perceptions.

PCA and HCA were performed by SIMCA‐P 13.0 software (Umetrics, Umeå, Sweden). ANOVA was performed by SPSS 20.0 software (SPSS Inc., Chicago, IL, USA). PLSR was performed by XLSTAT 2014 (Addin soft, Paris, France). The aroma profile plot, aroma interaction plot, and heatmap were obtained using Origin 9.1 (Origin Lab, Northampton, MA).

## 3 RESULTS AND DISCUSSION

### 3.1 Changes in sensory properties of BGT during roasting

The main sensory attributes of BGT include “fresh,” “flower,” “tender,” “sweet,” and “chestnut‐like.” To better understand changes in sensory attributes of BGT during roasting, HCA and PCA were performed to visualize differences in sensory attributes of BGT samples collected during roasting.

**FIGURE 1:**
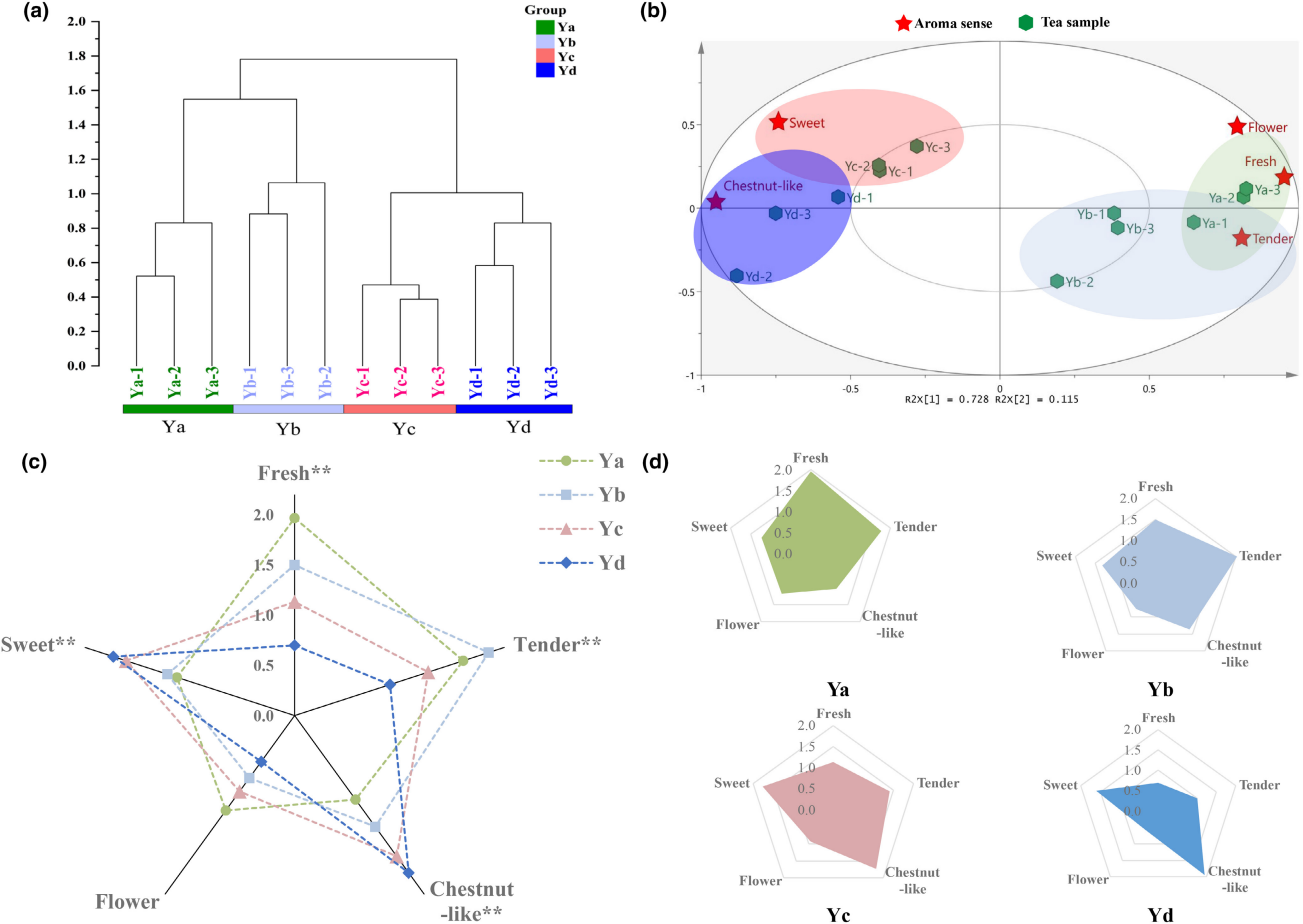
Changes in aroma sensory during BGT roasting process. (a) Hierarchical cluster analysis (HCA) plot of BGT aroma sensory profiles at different roasting stages; (b) Principal component analysis (PCA) plot of BGT aroma sensory profiles at different roasting stages. (c) Comparison of Aroma Sensory Radar of BGT B at different roasting stages, *significant differences (*p* < .05), **extremely significant differences (*p* < .01). (d) Aroma Sensory Radar of BGT at different roasting stages. All Ya, Yb, Yc and Yd in the figure represent tea samples from different roasting stages (0, 15, 50 and 75 min), and the number after the abbreviation represents repetition

HCA results (Figure 1a) showed that the four samples clustered into four categories; in particular, Ya and Yb clustered relatively close, as well as Yc and Yd, thus indicating that aroma profiles of BGT samples underwent significant and gradual changes during roasting, of which process Ya and Yb may belong to the first stage, and Yc and Yd to the second stage. Furthermore, PCA results (Figure 1b) showed that PC1 and PC2 contributed to explaining 75.4% and 12.0% of total variance in the data, respectively, with the first two components explaining 87.4% of total variance in the data. Notably, BGT samples at different roasting stages were better distributed along PC1, in which samples Ya and Yb were located on the right side of the vertical axis, which was positively correlated with the odor descriptors such as “fresh,” “flower,” and “tender.” Conversely, samples Yc and Yd were distributed on the left side of the axis, and positively correlated with “sweet” and “chestnut‐like” odor descriptors. Therefore, PCA results were in agreement with HCA results. In general, according to the sensory descriptors used in PCA and HCA, it can be stated that sensory changes in BGT during roasting occurred in two stages: (i) stage I/Ya and Yb stage, during which BGT aroma quality was dominated by “fresh,” “flower,” and “tender” attributes; (ii) stage II/Yc and Yd stage, during which BGT aroma was mainly characterized by “sweet” and “chestnut‐like.”

Subsequently, in order to better understand the sensory characteristics of BGT samples at different stages of the roasting process, an aroma sensory radar was used (Figure 1c,d), which showed differences in sensory characteristics of tea leaves at different stages of the roasting process. Figure 1c shows that except for the “flower” descriptor, the descriptors “fresh,” “tender”, “chestnut‐like,” and “sweet” changed significantly during roasting of BGT (*p* < .01). Figure 1d shows that the descriptors “fresh” and “tender” were the strongest in the Ya stage, while “chestnut‐like” was the weakest; the descriptors “tender” and “flower” were the weakest in the Tb stage; the descriptors “sweet” and “chestnut‐like” were the strongest in the Yc stage, whereas “fresh” was the weakest; similar to Yc stage, the descriptor “chestnut‐like” was the strongest in Yd stage, whereas “fresh” was the weakest. Overall, by prolonging the duration of roasting, BGT aroma gradually changed from “fresh” to “tender,” and finally, to “sweet” and “chestnut‐like.”

### 3.2 Changes in volatile compounds of BGT during roasting

#### 3.2.1 Overall changes in volatile compounds

HS‐SPME‐GC–MS was used to determine volatile compounds in BGT during roasting. A total of 313 volatile compounds were identified in samples (Figure S1), including 68 esters, 65 terpenes, 58 heterocyclic compounds, 36 alcohols, 33 ketones, 29 aldehydes, 14 aromatics, 5 phenols, 4 acids, and 1 sulfide.

**FIGURE 2:**
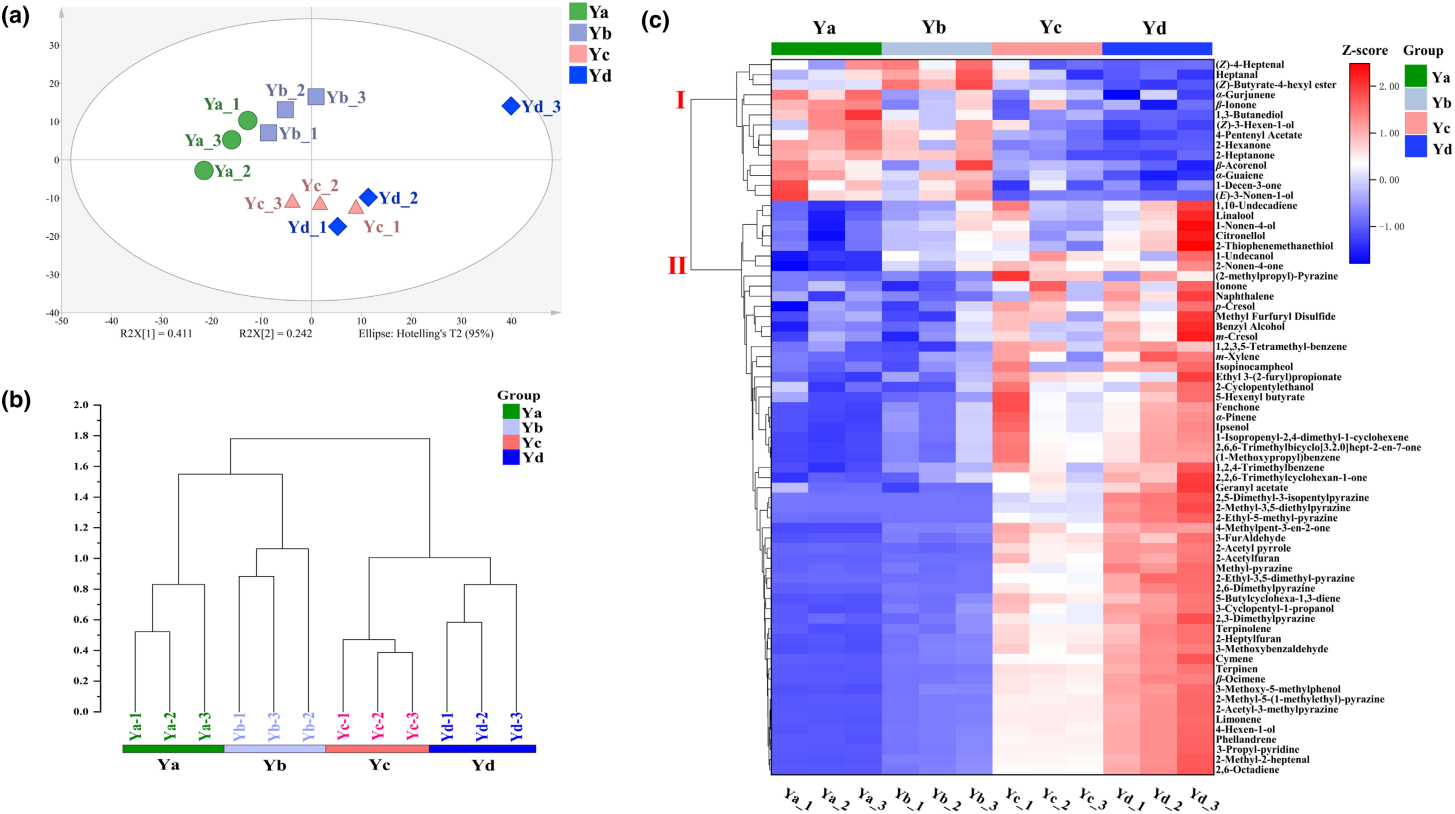
Changes in volatile compounds during BGT roasting. (a) PCA plot of BGT aroma compound contents at different roasting stages. (b) HCA plot of BGT aroma compound contents at different roasting stages. (c) Heatmap of key volatile compounds during BGT roasting (*p* < .05).

Thus, to better understand overall changes in volatile compounds in BGT during roasting, PCA and HCA were used to visualize differences in volatile compounds among samples collected throughout the roasting process. PCA results are shown in Figure 2a, PC 1 and PC 2 explained 41.1% and 24.2% of total variance in the data, respectively, with the first two components explaining 65.3% of total variance. Overall, HCA results showed consistency with PCA results. As shown in Figure 2b, HCA plot revealed that the four samples clustered into two categories, with Ya and Yb forming one category, and Yc and Yd the other category. Overall, according to changes in volatile compounds in BGT, it can be stated that roasting occurred in two stages: stage (I) Ya and Yb stage and stage (II) Yc and Yd stage, which is consistent with the results observed for aroma profiles discussed above (Figure 1), thus indicating that the volatile compounds identified herein largely explain the changes in sensory attributes of BGT during roasting.

#### 3.2.2 Specific changes in volatile compounds of BGT during roasting

A total of 72 significantly different volatile compounds (*p* < .05) were identified among 313 volatile compounds, which can be considered as key substances to distinguish tea samples from different baking stages. The heatmap and HCA results of these 72 volatile compounds are shown in Figure 2c.

As shown in Figure 2c, the 72 volatile compounds were clustered into two groups. The concentrations of volatile compounds in group I decreased significantly during roasting, which included 14 volatile compounds, mainly terpenes (*β*‐ionone), alcohols [(*Z*)‐3‐hexen‐1‐ol and 1,3‐butanediol], and aldehydes [(*Z*)‐4‐heptenal and heptanal]. It is known that these volatile compounds and precursors may undergo further volatilization, oxidation, degradation, and esterification during roasting, thus leading to a significant decrease in their concentrations (Lei et al., 2017; Yang et al., 2021). Previous studies showed that short‐chain unsaturated alcohols and aldehydes confer a typical aroma resembling freshly cut grass or crushed green plant leaves (i.e., grass smell) (Akacha & Gargouri, 2009; Yang, 2013). Thus, a decrease in the abundance of these volatile compounds may be the main reason for the reduction in “fresh” and “tender” sensory characteristics in BGT during roasting. Moreover, the decrease in the concentration of *β*‐ionone (strong floral and fruity aroma) may be attributed to the decrease in the perception of “flower” aroma during roasting of BGT. Studies have shown that, after thermal processing, (*Z*)‐3‐hexen‐1‐ol in tea volatilizes in large quantities, resulting in changes in tea aroma from “smelly” to “fresh,” which eventually fades (Yin et al., 2022). The former stage mostly occurs during the fixing step of fresh leaves, and the latter stage occurs during drying and roasting. Furthermore, the decrease in the abundance of other short‐chain unsaturated alcohols and aldehyde could be mostly due to thermal degradation (Revichandran & Parthiban, 2000).

There are 58 volatile compounds in Group II, and their concentrations significantly increase during the roasting process. Combined with the sensory changes in BGT during roasting, we speculate that these compounds are closely related to the formation of chestnut‐like aroma in BGT. These compounds can be divided into two categories based on their aroma properties. The first category includes sweet floral compounds such as linalool, benzyl alcohol, ionone, and ethyl salicylate, which mainly come from the hydrolysis of glycoside precursors and the oxidative degradation of carotenoid substances in tea, and have high boiling points (Feng et al., 2019; Ho et al., 2015; Yang, 2013). The second category includes caramel‐like compounds such as 2,6‐dimethylpyrazine, 2,3‐dimethylpyrazine, 2‐ethyl‐3,5‐dimethylpyrazine, 2‐acetylpyrrole, 3‐furfural, and 2‐acetylfuran, which mostly come from the Maillard reaction between amino acids and sugars at high temperature (Boekel, 2006). These aroma compounds have been reported as key compounds for chestnut aroma in green tea in different studies (; Wang et al., 2020; Zhu et al., 2018). Almost none of the 58 aroma compounds mentioned above have a chestnut aroma. Based on previous research, we infer that the formation of chestnut aroma in green tea is not determined by one or a group of compounds, but rather by the collective action of compounds with different aroma types. In this study, it was confirmed that floral and caramel‐like compounds participate in the formation of chestnut aroma in BGT. Caramel‐like compounds may be the main contributors to the chestnut aroma, while floral compounds may play a regulatory role. However, this is our hypothesis, and the formation mechanism of chestnut aroma in tea is similar to the “intestinal microbiota,” involving the interaction of various aroma compounds in tea. This mechanism requires further in‐depth research.

Furthermore, it is worth noting that when the concentration of pyrazine aroma compounds produced by the Maillard reaction is too high, it can cause burnt odor in tea. However, in this study, no strong burnt odor was detected in BGT, which may be related to the low‐temperature roasting process used. In future research, different roasting temperatures may be set to explore the relationship between roasting temperature and burnt odor in tea.

### 3.3 Relationship between aroma profiles and volatile compounds in BGT during roasting

**FIGURE 3:**
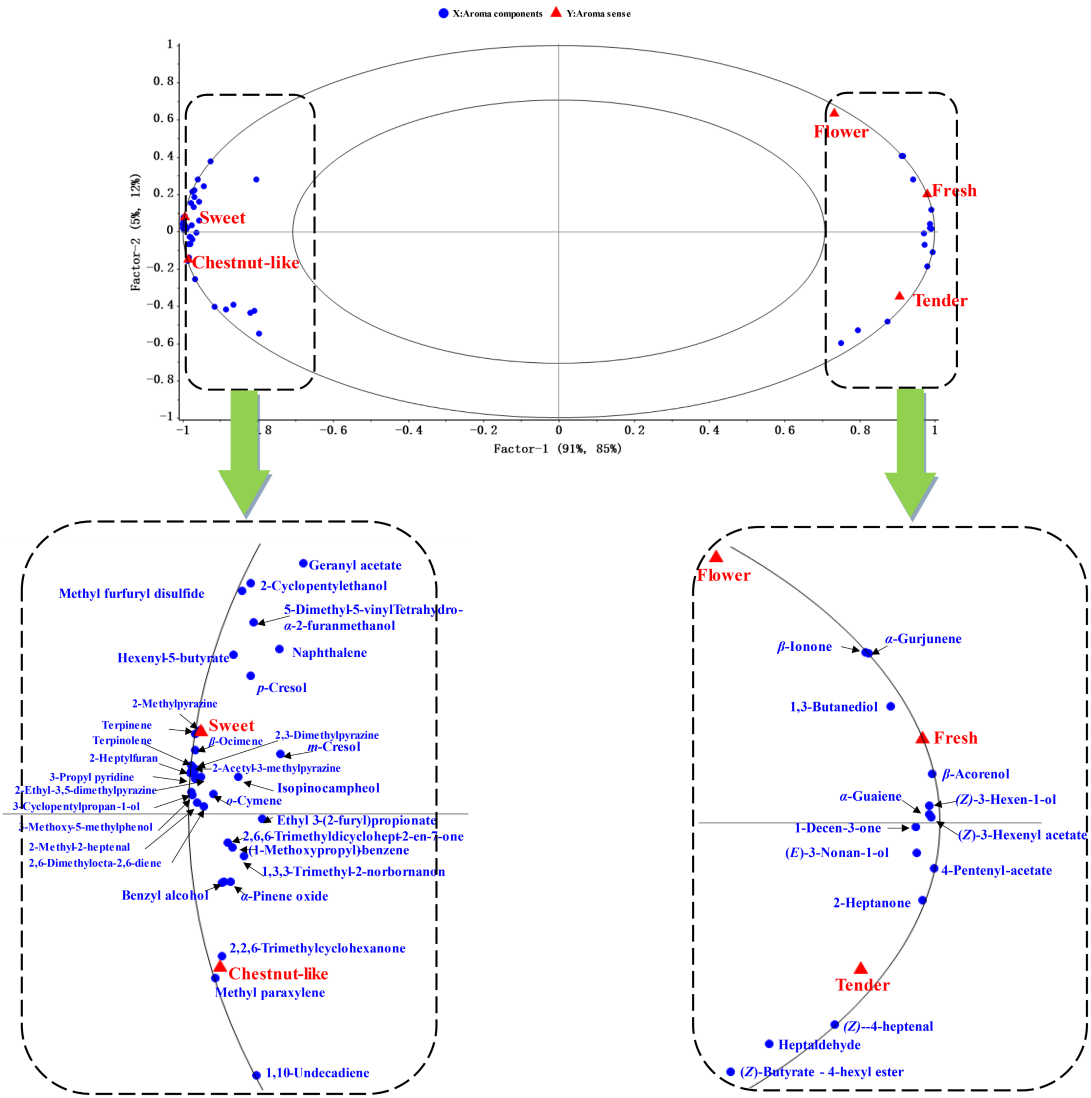
PLSR plot of the relationship between the aroma sensory and aroma components during BGT roasting. Blue dots are volatile compounds (independent variable x, *n* = 72), and red triangles are aroma senses (dependent variable y, *n* = 5), the volatile compounds plotted in the vicinity of the aroma sensory attributes were positively associated with those attributes.

PLSR analysis was used to determine the relationship between aroma sensory profiles and volatile compounds in BGT during roasting (Figure 3). In Figure 3, most of the volatile compounds and sensory descriptor points were distributed in the outer circle (*R* ^2^ = 1) of the PLSR model, thus indicating that it was acceptable.

From Figure 3, it can be seen that there are two main types of aroma compounds that contribute significantly to the freshness of BGT. One type is represented by fatty alcohols such as (*Z*)‐3‐hexen‐1‐ol and (*E*)‐3‐nonanol, which are derived from the oxidation of long‐chain fatty acids in tea (Heilmann, 2009). These alcohols are considered to be the main contributors to the grassy aroma in tea (Yin et al., 2022). (*Z*)‐3‐hexen‐1‐ol has a low boiling point and is a volatile short‐chain alcohol, so the volatilization of (*Z*)‐3‐hexen‐1‐ol during the roasting process of BGT may be the main reason for the decrease in the fresh sensory perception of BGT, which is consistent with our previous speculation. The other class of compounds that contribute significantly to the fresh aroma are ester compounds represented by (*Z*)‐3‐hexenyl acetate and butyl 4‐hydroxyhexanoate. These esters are mainly formed by the reaction of the aforementioned fatty acid oxidation products with acetic acid (Feng et al., 2019). They are a common type of volatile component in green tea with a fresh odor (Flaig et al., 2020). (*Z*)‐3‐Hexenyl acetate is formed by the esterification of (*Z*)‐3‐hexen‐1‐ol with acetic acid. It has a strong grassy odor itself and a low boiling point. It is also easily volatilized during the baking process, leading to a decrease in its concentration and thus a decrease in the fresh sensory perception of BGT.

Notably, the “flower” aroma is relatively rare in green tea, with only certain high‐quality green tea types made from fresh leaves grown in a few regions showing an orchid fragrance. PLSR results obtained herein showed that the relative low abundance of volatile compounds related to the “flower” aroma, with only *β*‐ionone and *α*‐gurjunene contributing to “flower” aroma in BGT. *β*‐Ionone mainly comes from the degradation of carotenoids during the heating process of green tea (Ho et al., 2015). Due to its strong floral aroma and low threshold (0.007 μg/kg), *β*‐ionone has been identified as an important compound contributing to the floral aroma of green teas (Baba et al., 2017; Wang, Dai, et al., 2022; Wang, Du, et al., 2022), which is consistent with the results of this study. *α*‐Gurjunene belongs to the terpene compounds and has a “wood balsam” aroma itself, so it may contribute to the floral aroma of BGT. Currently, most reports on *α*‐gurjunene are focused on its functional activities (Cunico et al., 2007; El Ghorab et al., 2002; Hazzit et al., 2006), and there are relatively few related reports on its aroma contribution in tea.

In addition, the volatile compounds that contributed greatly to the “tender” quality were mainly 2‐heptanone, (*Z*)‐4‐heptenal, and heptanal, which are considered fatty aldehydes. Lipoxygenase‐mediated lipid oxidation is the main pathway by which these key odorants are formed (Ho et al., 2015). In the sensory evaluation, the BGT sample that scored higher in “tender” was Yb, and the sample was at early stage of roasting, in which the content of fatty aldehydes generated by lipid degradation was the highest, which mostly confer the “tender” aroma quality.

Furthermore, the volatile compounds that contributed greatly to generating the “sweet” and “chestnut‐like” attributes were mainly 2‐ethyl‐3,5‐dimethylpyrazine, 2,3‐dimethylpyrazine, 2‐acetyl‐3‐methylpyrazine, 2‐methylpyrazine, and 2‐heptylfuran. Pyrazines are a product of the Maillard reaction occurring between sugars and amino acids and are associated with “coconut”, “toasty”, and “caramel” sensory attributes. In contrast, furans are thought to confer “almond” and “caramel‐like” sensory attributes (Fan et al., 2007). Buttery et al. (1973) found that 2‐ethyl‐3,5‐dimethylpyrazine was the key compound conferring the “bakery” aroma in potatoes. Gong et al. (2017) used GC‐O and OAV experiments to determine the active aroma substances in three types of Longjing tea, which showed that 2‐ethyl‐3,5‐dimethylpyrazine was the active aroma compound in roasted Longjing tea, which is consistent with the results obtained herein. In addition, combined with the heatmap shown in Figure 2c, the key to distinguishing the “sweet” and “chestnut‐like” aroma attributes in tea was the overall concentration of Maillard reaction products (e.g., pyrazines) rather than the diversity of volatile compounds species, with the products of Maillard reaction in “chestnut‐like” tea samples found at higher concentrations.

### 3.4 The sensory interaction between different aroma types in BGT

**FIGURE 4:**
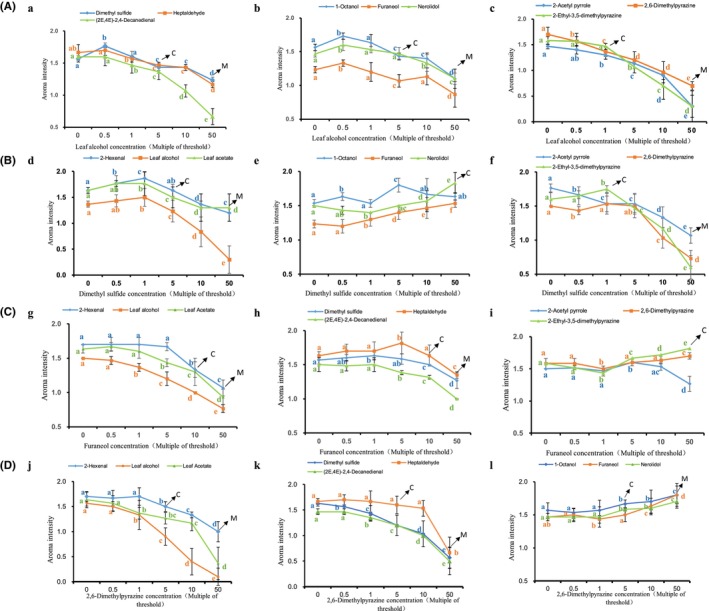
The interaction effect plots between different types of volatile compounds. (A) The effect of “fresh” on other aroma. (B) The effect of “tender” on other aroma. (C) The effect of “sweet” on other aroma. (D) The effect of “chestnut‐like” on other aroma. “a”. Effect of “fresh” on “tender” attribute. “b”. Effect of “fresh” on “sweet” attribute. “c”. Effect of “fresh” on “chestnut‐like” attribute. “d”. Effect of “tender” on “fresh” aroma attribute. “e”. Effect of “tender” on “sweet” aroma attribute. “f”. Effect of “tender” on “chestnut‐like” aroma attribute. “g”. Effect of “sweet” on “fresh” aroma attribute. “h”. Effect of “sweet” on “tender” aroma attribute. “i”. Effect of “sweet” on “chestnut‐like” aroma attribute. “j”. Effect of “chestnut‐like” on “fresh” aroma attribute. “k”. Effect of “chestnut‐like” on “tender” aroma attribute. “l”. Effect of “chestnut‐like” on “sweet” aroma attribute. “C”. Change, represents the change of aroma types; “M”, Mask, represents the masking effect of aromas.

During roasting of BGT, the diversity of aroma attributes changed. To better understand the interaction between different aroma attributes, an in vitro interaction simulation was conducted. Since “flower” was not the main aroma attribute in BGT during roasting, the interaction experiment was aimed at elucidating the other four aroma attributes, that is, “fresh,” “tender,” “chestnut‐like,” and “sweet” found in BGT. The sensory interaction results between different aroma types in BGT are shown in Figure 4.

The interactions between different aroma attributes differed and included mutual promotion, mutual inhibition, and masking effects. In addition, the concentration of most of the volatile compounds that caused mutual transformation was five times the threshold concentration. In contrast, volatile compounds that caused the masking effect were found at concentrations nearly 50 times that of the threshold concentration.

Figure 4A depicts the effects of “fresh” aroma attribute on the other attributes (i.e., “tender,” “sweet,” and “chestnut‐like”). Overall, with the increase in the concentration of “fresh” attribute, an inhibitory effect was observed on the other aroma attributes, meaning that a high concentration of “fresh” attribute could inhibit “tender,” “sweet,” and “chestnut‐like” attributes. By contrast, at low concentrations (0.5 times that of the threshold concentration), the “fresh” attribute enhanced the performance of “tender” and “sweet” attributes, which may be due to the fact that, when the aroma compound related to the “fresh” attribute (i.e., (*Z*)‐3‐hexen‐1‐ol) is found at low concentrations, an aroma similar to green apples is produced, which likely is a reminiscent of “tender” and “sweet” aroma attributes. In addition, at five times the threshold concentration, “fresh” led to changes in “tender” and “sweet” attributes, while changes in “chestnut‐like” aroma occurred even when the threshold concentration was exceeded by one time. Taken together, the impact of “fresh” on “chestnut‐like” was greater than that on the other aromas, and a lower concentration of “fresh” aroma could lead to changes in the perception of “chestnut‐like” aroma.

Figure 4B shows the effects of “tender” aroma on “fresh,” “sweet,” and “chestnut‐like” aroma attributes. Unlike “fresh,” “tender” did not show an inhibitory effect on all other aroma types. Within the concentration range adopted in our experiment (0.5–50 times the threshold concentration), “tender” showed a promoting effect on “sweet” aroma attribute. In addition, at low concentrations (0.5–1 times), “tender” promoted the strength of “fresh” aroma attribute, which was weakened as the concentration of “tender” increased. In general, “tender” aroma plays an important role in green tea aroma, since it is considered the main aroma type of green tea and an important regulatory aroma type that enhance the performance of other aroma types, thereby enriching overall aroma quality of green tea.

It was shown that the effects of “sweet” and “chestnut‐like” on other aroma types were largely similar (Figure 4C,D); that is, low concentrations of “sweet” and “chestnut‐like” had almost no effect on the other aroma types, while high concentrations of “sweet” and “chestnut‐like” inhibited “fresh” and “tender” aroma. In addition, “sweet” and “chestnut‐like” mutually promoted aroma intensity.

The sensory interactions between different aroma types in BGT suggest strong inhibitory/masking/promoting interactions among different volatile aroma components, and this phenomenon has also been found in multiple other studies (Nie et al., 2020; Schuh & Schieberle, 2006). There may be three main mechanisms for this interaction: the chemical reactions of the aroma components themselves; the influence of one aroma substance on the signal transduction of another aroma substance at the olfactory receptor level; and one aroma substance has an impact on another aroma substance at the cognitive level of the cerebral cortex. The interaction results can be manifested as aroma synergy, aroma addition, aroma suppression, and aroma masking, which not only depend on the characteristics of the aroma substance itself but also on the concentration of the two substances when mixed (Guadagni et al., 1963; Hattori et al., 2005; Ito & Kubota, 2005). This requires us to design more in‐depth experiments for further exploration.

## 4 CONCLUSION

Herein, HS‐SPME‐GC–MS combined with QDA was employed to characterize volatile compounds and aroma sensory changes in BGT during low temperature roasting for the first time. The sensory perception of BGT gradually changed from “fresh” and “tender” to “sweet” and “chestnut‐like” during low‐temperature roasting, which was mainly due to the volatilization and degradation of short‐chain saturated alcohols and aldehydes, such as (*Z*)‐3‐hexen‐1‐ol and (*Z*)‐4‐heptenal, as well as the accumulation of furan and pyrazine volatile compounds, such as 2‐ethyl‐3,5‐dimethylpyrazine and 2‐acetylfuran. Moreover, in vitro interaction simulation assays showed that low concentrations of “fresh” and “tender” aroma attributes showed a mutual promotion effect; however, when the concentrations were high, these aroma types inhibited one another; of note, “sweet” and “chestnut‐like” generally showed a mutual promotion effect. These findings provide new insights into the changes in volatile metabolites in BGT during low‐temperature roasting.

In future research, in order to further explore the effects of different roasting temperatures on BGT, studies will consider increasing the range of roasting temperatures, including middle and high temperatures, to investigate the impact of these temperatures on the aroma of BGT. Absolute quantitative methods will be employed, combined with techniques such as odor activity value (OAV) and aroma extract dilution analysis (AEDA), to further explore the key aroma‐active substances at different roasting temperatures.

## AUTHOR CONTRIBUTIONS

**Cong‐Ming Wang:** conceptualization, formal analysis, investigation, and writing—original draft. **Xiao‐Qin Tan:** data curation, investigation, and writing—review & editing. **Xiao Du:** conceptualization, funding acquisition, and supervision. **Jia‐Jing Hu:** investigation and data curation. **Xin‐Yi Li:** investigation and data curation. **Li‐Shu Yan:** investigation. **Xiang Zhang:** methodology. **Cong‐Ning Nie:** methodology. **Liu‐Yi Chen:** software. **Feng Du:** investigation. **Yue‐Ling Zhao:** writing—review & editing. **Jin‐Lin Bian**: writing—review & editing. **Pin‐Wu Li:** conceptualization, funding acquisition, and writing—review & editing. All of the authors read and approved the final manuscript.

## CONFLICT OF INTEREST STATEMENT

The authors declare no competing interests.

## ETHICS STATEMENT

In the sensory evaluation part of this study, trained evaluators were used to conduct necessary sensory evaluations on tea samples. Except for the above parts, this study does not include other work with humans or animal. All methods were carried out in accordance with relevant guidelines and regulations and informed consent was obtained from all subjects before their participation in the study. No coercion to participate, full disclosure of study requirements and risks, no release of participant data without their knowledge, and ability to withdraw from the study at any time. All plants in this study comply with international, national, and/or institutional guidelines.

## Supporting information


Appendix S1.

## Data Availability

The authors declare that the data supporting the findings of this study are available within the article. The raw/derived data supporting the findings of this study are available from the corresponding author upon reasonable request.
