# Supplementary material for: Dynamic changes and interaction between different aroma types during low‐temperature roasting of bud green tea
Source: Food Sci Nutr. 2024 Jun 17;12(9):6367–78. doi: 10.1002/fsn3.4249 (PMC11561774; doi:10.1002/fsn3.4249)
Supplement: Supplementary file 1 — Appendix S1. [file FSN3-12-6367-s001.docx]

**Table S1. The making process of bud green tea before roasting**

| **Process** | **Describe** |
| --- | --- |
| Spreading | After picking fresh leaves, they are placed in a spreading tank. The thickness of the fresh leaves is 3cm, and the spreading time is 9h. After spreading, the water content of the tea leaves is about 70 ± 5% |
| Fixing | Fixing（called sha qing in Chinese）, which step that enzyme inactivation was achieved at high temperatures and short time, using a rotary continuous fixation machine. The fresh leaf tenderness was single bud. 6CSQ-60（Changsha Xiang feng Tea Machinery Manufacturing Co., Ltd） fixing machine was used, and the leaf dosage was 4.0 kg/min. The temperature of the machine was set at 280 ℃, the roller speed was set at 28 r / min, and the fixing time was 120 ± 5 S. After fixing, the moisture content of the leaf was about 55 ± 5 %. |
|  |  |
| Shaping | Tea leaves swing rapidly in a certain space through a special shaping machine. This step can make the tea shape straight and even. 6CCB-50 （Changsha Xiang feng Tea Machinery Manufacturing Co., Ltd）shaping machine was adopted, the temperature was set at 110 ℃, rotating speed 190r / min, leaf volume 2.0kg/pot, shaping time 35 ± 2min。 After shaping, the leaves are flat and straight, the color was green, and the moisture content was about 20 ± 5 %. |
|  |  |
|  |  |
|  |  |
| Drying | 6CH-16（Changsha Xiangfeng Tea Machinery Manufacturing Co., Ltd） drying machine was used, the temperature was set at 100 ℃ and the time was 20 ± 2 min. After drying, the moisture content of tea was 6 ± 1 %. |

**Table S2. The basis for the selection of chemical reference standards in aroma sensory analysis and the interpretation of the aroma**

| **Aroma Sensory** | **Descriptiona** | **Reference standard concentration** | **Intensity scoring criteria** |
| --- | --- | --- | --- |
| Fresh | Clean and refreshing, aroma similar to grass or fresh leaves | (*Z*)-3-hexenol | 0 points: Reference standard concentration OAV=0;  0.5 points: Reference standard concentration OAV=5^0^;  1 point: Reference standard concentration OAV=5^1^;  1.5 points: Reference standard concentration OAV=5^2^;  2 points: Reference standard concentration OAV=5^3^;  2.5 points: Reference standard concentration OAV=5^4^;  3 points: Reference standard concentration OAV=5^5^; |
| Tender | The pleasant and delicate aroma of tender raw materials, aroma similar to that of tender corn | Nonanal |  |
| Chestnut-like | The aroma is like cooked chestnut | 3-Methylbutyraldehyde |  |
| Sweet | Aroma as heated brown sugar | Phenylethanol |  |
| Flower | It evokes a pleasant fragrance of fresh flowers, such as rose fragrance, orchid fragrance, etc | Linalool |  |

a Descriptors references national standards: GB / T 14487-2017 tea vascular for sensory evaluation.

**Table S3. OAV evaluation results of volatile compounds of bud green tea in different roasting stages**

| **Number** | **Volatile compounds** | **Retention indices** | **CAS** | **threshold（mg/kg）** | **OAV^a^** | | | | **Description of aroma (FEMA/ Chemical Book)** |
| --- | --- | --- | --- | --- | --- | --- | --- | --- | --- |
|  |  |  |  |  | **Ya** | **Yb** | **Yc** | **Yd** |  |
| 1 | Linalool | 1099 | 78-70-6 | 0.001500 | 5499.77（1/IV） | 6241.25（1/III） | 6366.18（1/II） | 6898.78（1/I） | Coriander, floral, lavender, lemon, rose |
| 2 | (*E*)-2-Nonenal | 1162 | 18829-56-6 | 0.000400 | 3398.99 （2/IV） | 3559.10（2/III） | 3668.68 （2/II） | 3720.01 （2/I） | Aroma of sweet, baking, bread, cooking, fruit and caramel |
| 3 | (*Z*)-2-Butenoic acid ethyl ester | 830 | 6776-19-8 | 0.000060 | 1238.41 （3/II） | 1230.48（4/III） | 1244.38（4/I） | 1157.32（4/IV） | Intense aromas of apple, pineapple and unripe plum peel |
| 4 | (*E, E*)-2,4-decadien-1-al | 1317 | 25152-84-5 | 0.000070 | 1048.18 （4/IV） | 1565.58（3/II） | 1612.06 （3//I） | 1298.90 （3/III） | Aroma of orange, fresh sweet orange(Characteristic aroma components of black tea |
| 5 | 2,4-Dimethyl-1-(1-methylethenyl)- cyclohexene | 1108 | 56763-60-1 | 0.005000 | 665.57 （5/IV） | 798.31 （5/III） | 1014.65 （5/II） | 1065.75 （5//I） | Fresh fruit and cool camphor aromas, at high concentrations, it is smoky odor. It is produced in the process of tea fermentation and drying. |
| 6 | (*E, Z*)-2,4-Decadienal | 1295 | 25152-83-4 | 0.000160 | 541.90 （6/III） | 602.96（6//I） | 519.34（6/IV） | 554.48 （6/II） | Intense aroma of chicken and chicken fat |
| 7 | 5-Trimethyl-5-vinylfuran-2-methanol | 1086 | 34995-77-2 | 0.006000 | 157.71 （7/IV） | 176.68 （7/II） | 163.31 （7/III） | 179.11 （7//I） | Strong woody, floral, terpene, delicate, fruity aromas |
| 8 | Furaneol | 1070 | 3658-77-3 | 0.030000 | 111.65（8/IV） | 117.29 （8/III） | 123.37 （8/II） | 126.06 （8//I） | Aroma of sweet, baking, bread, cooking, fruit and caramel |
| 9 | (*E*)-2-Octenal | 1060 | 2548-87-0 | 0.003000 | 78.73 （9/II） | 81.30 （9//I） | 65.76 （10/IV） | 72.81 （10/III） | Fat and meat aromas, with hints of cucumber and chicken |
| 10 | 2,2,6-Trimethyl-6-vinyltetrahydro-2h-pyran-3-ol | 1173 | 14049-11-7 | 0.006000 | 71.18 （10/III） | 70.64 （10/IV） | 80.62 （9/II） | 91.14 （9//I） | It has a more obvious floral aroma, and a lower tea aroma |
| 11 | 1-Dodecanol | 1474 | 112-53-8 | 0.003500 | 45.58 | 38.09 | 47.14 | 46.86 | It has a strong fatty aroma with a strong aroma similar to pine leaf oil and orange oil |
| 12 | Benzyl acetate | 1162 | 140-11-4 | 0.002600 | 44.62 | 46.67 | 43.84 | 45.31 | It has a special aromatic smell similar to jasmine, bitter and spicy |
| 13 | Naphthalene | 1182 | 91-20-3 | 0.050000 | 31.41 | 31.84 | 33.39 | 34.32 | It has a special aroma |
| 14 | Citral | 1273 | 5392-40-5 | 0.032000 | 25.81 | 27.71 | 24.75 | 27.02 | It has a strong lemon odor |
| 15 | Methyl salicylate | 1192 | 119-36-8 | 0.060000 | 15.79 | 15.59 | 18.33 | 19.20 | Holly leaves fragrant, with a special smell of herbs |
| 16 | 2-N-Heptylfuran | 1195 | 3777-71-7 | 0.003600 | 15.69 | 21.28 | 41.63 | 53.22 | Green aroma, fat, greasy, lactone aroma, milk, pork aroma |
| 17 | Citronellol | 1228 | 106-22-9 | 0.003500 | 15.17 | 16.56 | 17.04 | 18.95 | Has similar special aroma of fresh rose |
| 18 | 2-Pentylfuran | 993 | 3777-69-3 | 0.004800 | 13.74 | 12.16 | 13.78 | 13.37 | The aroma of rum and bread |
| 19 | Hexanal | 801 | 66-25-1 | 0.005000 | 12.37 | 0.00 | 12.62 | 13.17 | Raw oil, grass and apple aromas |
| 20 | *α*-Pinene oxide | 1095 | 1686-14-2 | 0.120000 | 7.68 | 8.75 | 10.46 | 10.81 | Aromas of pinewood, needles and resin |
| 21 | (*Z*)-3,7-dimethylocta-2,6-dienal | 1240 | 106-26-3 | 0.030000 | 7.46 | 7.61 | 7.24 | 7.78 | It has a strong lemon odor |
| 22 | Phenethyl acetate | 1260 | 103-45-7 | 0.020000 | 6.86 | 7.23 | 7.65 | 8.32 | Has a sweet smell |
| 23 | (*Z*)-3-Hexenyl Acetate | 1009 | 3681-71-8 | 0.012100 | 5.18 | 0.00 | 0.00 | 0.00 | It has a strong aroma of new grass, green leaves and fruits |
| 24 | Hexyl hexanoate | 1384 | 6378-65-0 | 0.040000 | 4.85 | 4.54 | 4.64 | 4.84 | Aromas of green beans and raw fruit |
| 25 | *o*-cymene | 1022 | 527-84-4 | 0.013300 | 4.21 | 7.75 | 20.60 | 33.34 | A pleasant aroma |
| 26 | Heptaldehyde | 903 | 111-71-7 | 0.031000 | 3.97 | 4.47 | 3.75 | 3.58 | Aromas of fruits |
| 27 | 1-Octanol | 1099 | 111-87-5 | 0.054000 | 3.91 | 4.36 | 3.86 | 4.41 | Has dried sweet and sharp fatty wax aroma, with orange, orange peel, and the smell of roses samples. It also has a fruity, greasy, sweet and slightly grassy aroma. |
| 28 | 2-Ethyl-3,5-dimethylpyrazine | 1162 | 55031-15-7 | 0.001600 | 3.58 | 4.00 | 12.92 | 21.28 | The aroma of baking, baked potatoes, broth |
| 29 | (*Z*)-3-Hexen-1-ol | 830 | 928-96-1 | 0.013000 | 3.11 | 2.53 | 1.47 | 1.19 | Intense grass aromas and fresh tea notes |
| 30 | Nerolidol | 1317 | 7212-44-4 | 0.015000 | 2.95 | 3.26 | 3.28 | 3.52 | Sweet aromas of rose, lily of the valley and apple blossom |
| 31 | Benzaldehyde | 1108 | 100-52-7 | 0.300000 | 2.35 | 2.51 | 2.60 | 3.00 | Bitter almond, cherry and nut aromas |
| 32 | 1-Nonanol | 1295 | 143-08-8 | 0.086000 | 2.21 | 2.41 | 2.53 | 2.96 | Sweet and green rose wax and fruity fat wax aromas |
| 33 | Irisone | 1086 | 14901-07-6 | 1.080000 | 1.98 | 1.81 | 1.77 | 1.74 | Aromas of fruits, wood, fresh scent and violet |
| 34 | 2-Heptanone | 1070 | 110-43-0 | 0.006800 | 1.90 | 1.88 | 1.38 | 1.27 | Aromas of fruits fresh scent and nuts |
| 35 | 6,10-Dimethyl-5,9-undecadien-2-one | 1060 | 689-67-8 | 0.060000 | 1.77 | 1.56 | 1.95 | 2.00 | Fragrance, floral and fruity |
| 36 | 3-Methoxy-5-methylphenol | 1173 | 3209-13-0 | 0.050000 | 1.51 | 2.24 | 4.50 | 6.07 | The important aroma component of Longjing tea presents the aroma of cantaloupe |
| 37 | Butyl butyrate | 1474 | 109-21-7 | 0.100000 | 0.98 | 1.03 | 1.00 | 1.05 | It has aromas of apples |
| 38 | Geranyl butyrate | 1162 | 106-29-6 | 0.100000 | 0.75 | 0.82 | 0.77 | 0.84 | Light rose aromas and sweet apricot flavours |
| 39 | (*E*)-2-hexenal | 1182 | 6728-26-3 | 0.190000 | 0.70 | 0.72 | 0.79 | 0.63 | Intense aromas of fresh fruits and green leaves |
| 40 | Dipentene | 1273 | 5989-27-5 | 0.500000 | 0.47 | 0.84 | 2.26 | 3.18 | Pleasant fresh orange aroma, should be free of camphor and terpene |
| 41 | Benzyl alcohol | 1192 | 100-51-6 | 5.500000 | 0.19 | 0.20 | 0.21 | 0.23 | Sweet, floral, fruity, with a slight aroma |
| 42 | 1-Heptanol | 1195 | 111-70-6 | 0.700000 | 0.13 | 0.14 | 0.16 | 0.15 | Has oil flavor and spicy aromas, approximate citrus aromas |
| 43 | 2-Acetyl-3-methylpyrazine | 1228 | 23787-80-6 | 10.82000 | 0.01 | 0.01 | 0.04 | 0.06 | The aroma of roasted peanuts and meat |
| 44 | *m*-Xylene | 993 | 108-38-3 | 3.000000 | 0.04 | 0.04 | 0.05 | 0.05 | Have a fragrant smell |
| 45 | 1-Pentanol | 801 | 71-41-0 | 5.000000 | 0.03 | 0.03 | 0.03 | 0.03 | Natural fruit aroma |
| 46 | *o*-Xylene | 1095 | 95-47-6 | 3.000000 | 0.02 | 0.02 | 0.02 | 0.03 | Have a fragrant smell |
| 47 | 3-Furaldehyde | 1240 | 498-60-2 | 3.000000 | 0.01 | 0.02 | 0.06 | 0.07 | Have a special fragrant smell |
| 48 | Pyrazine, 3,5-diethyl-2-methyl- (8CI,9CI) | 1260 | 18138-05-1 | 0.820000 | 0.01 | 0.01 | 0.04 | 0.11 | Similar to whiskey and roasted peanuts aroma |
| 49 | 2,2,6-Trimethylcyclohexanone | 1009 | 2408-37-9 | 40.00000 | 0.00 | 0.00 | 0.01 | 0.01 | It has an earthy smell |
| 50 | 2-Acetyl pyrrole | 1384 | 1072-83-9 | 17.00000 | 0.00 | 0.00 | 0.02 | 0.03 | Aromas of dried fruit, licorice and toasted bread. It's the main component of the caramel aroma |
| 51 | 2,3-Dimethylpyrazine | 1022 | 5910-89-4 | 10.82000 | 0.00 | 0.00 | 0.01 | 0.01 | The aroma of roasted peanuts and meat |
| 52 | 2-Acetylfuran | 903 | 1192-62-7 | 80.00000 | 0.00 | 0.00 | 0.02 | 0.03 | Aromas of almond, nut, yeast, milk and sweet caramel |
| 53 | 1,2,4-Trimethylbenzene | 1099 | 95-63-6 | 0.058000 | 0.00 | 1.11 | 1.34 | 1.52 | Has a special aroma |
| 54 | 1-Hexanol | 1162 | 111-27-3 | 0.250000 | 0.00 | 0.37 | 0.32 | 0.23 | Soft green aromas of young branches and leaves with hints of wine, fruit and fat. |
| 55 | Geranyl acetate | 830 | 105-87-3 | 0.100000 | 0.00 | 0.00 | 0.73 | 0.88 | Rose oil and lavender oil mixed like aroma, diluted to apple aroma |
| 56 | *α*-Copaene | 1317 | 3856-25-5 | 0.120000 | 0.00 | 0.00 | 0.46 | 0.51 | Aromas like ionone.It has the smell of pine, needles and resin |

Arabic numerals in brackets are sorted by OAV size in vertical column, and Roman numerals are sorted by OAV size in horizontal column.

^a^OAV: the ratio of Volatile compounds to their threshold

| 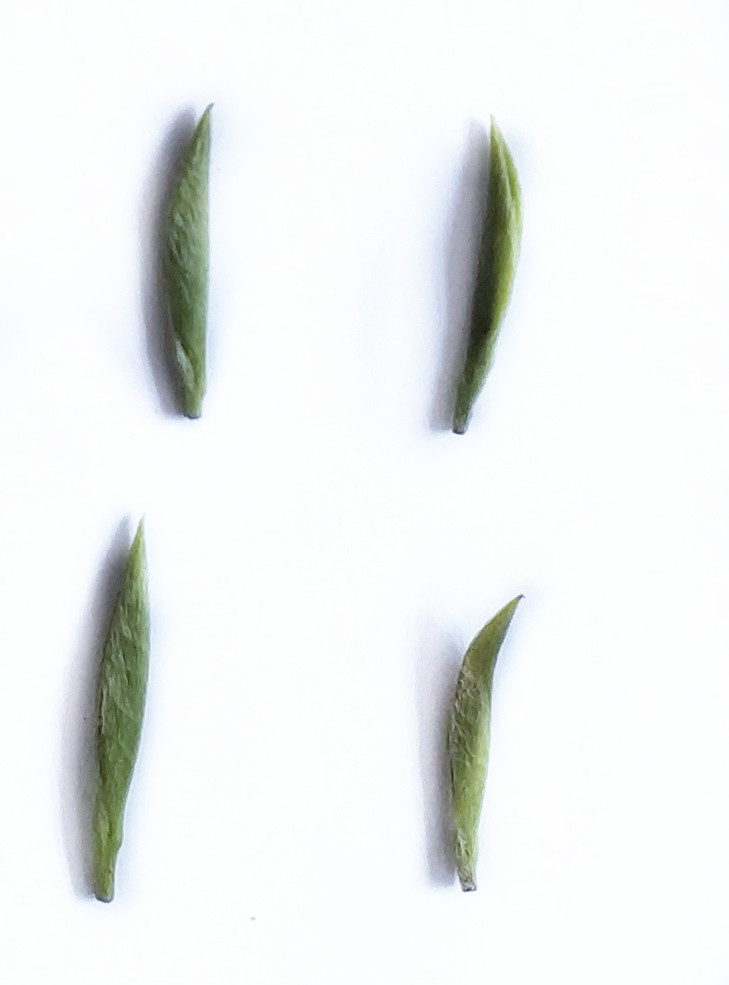 | 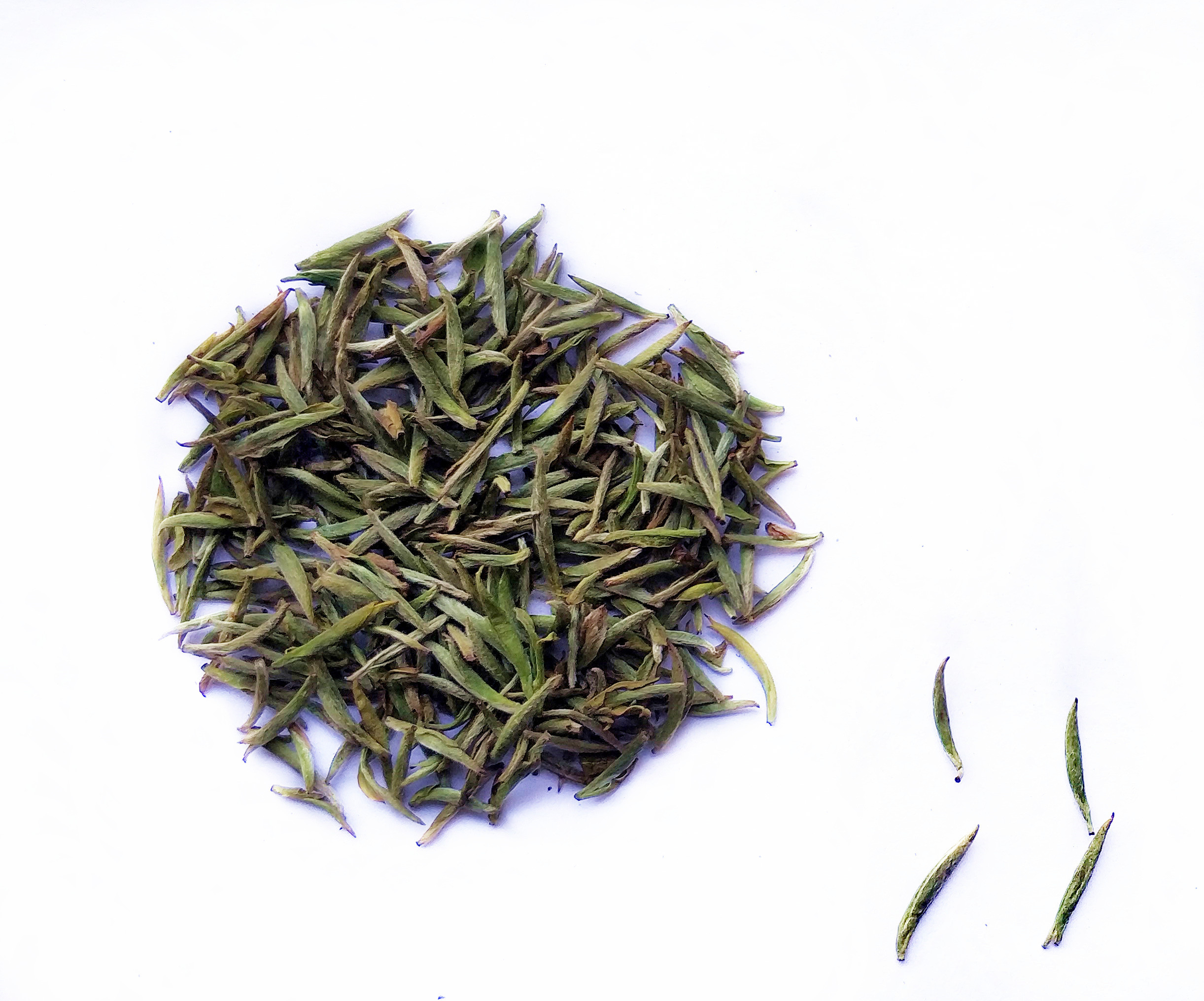 | 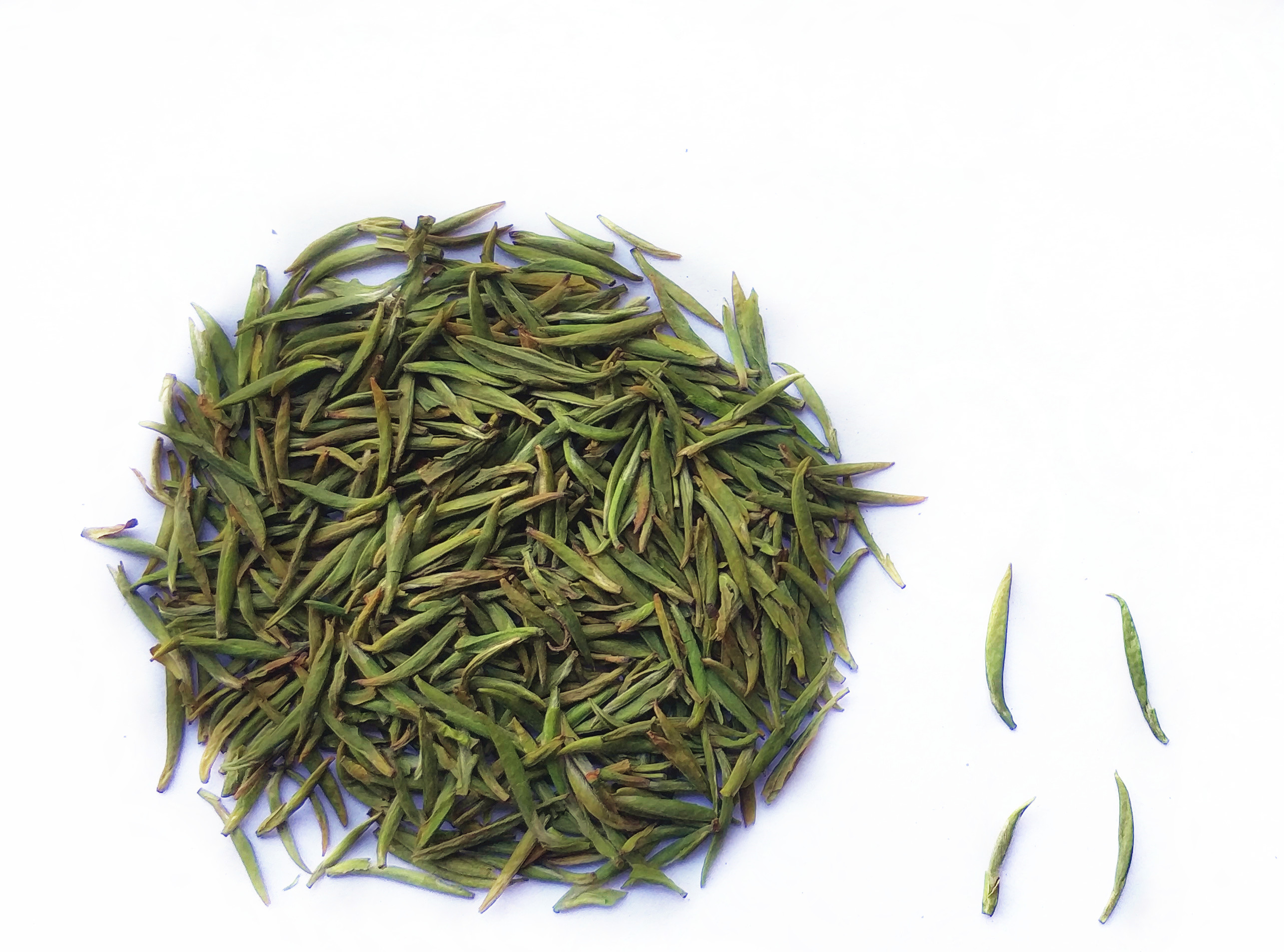 | 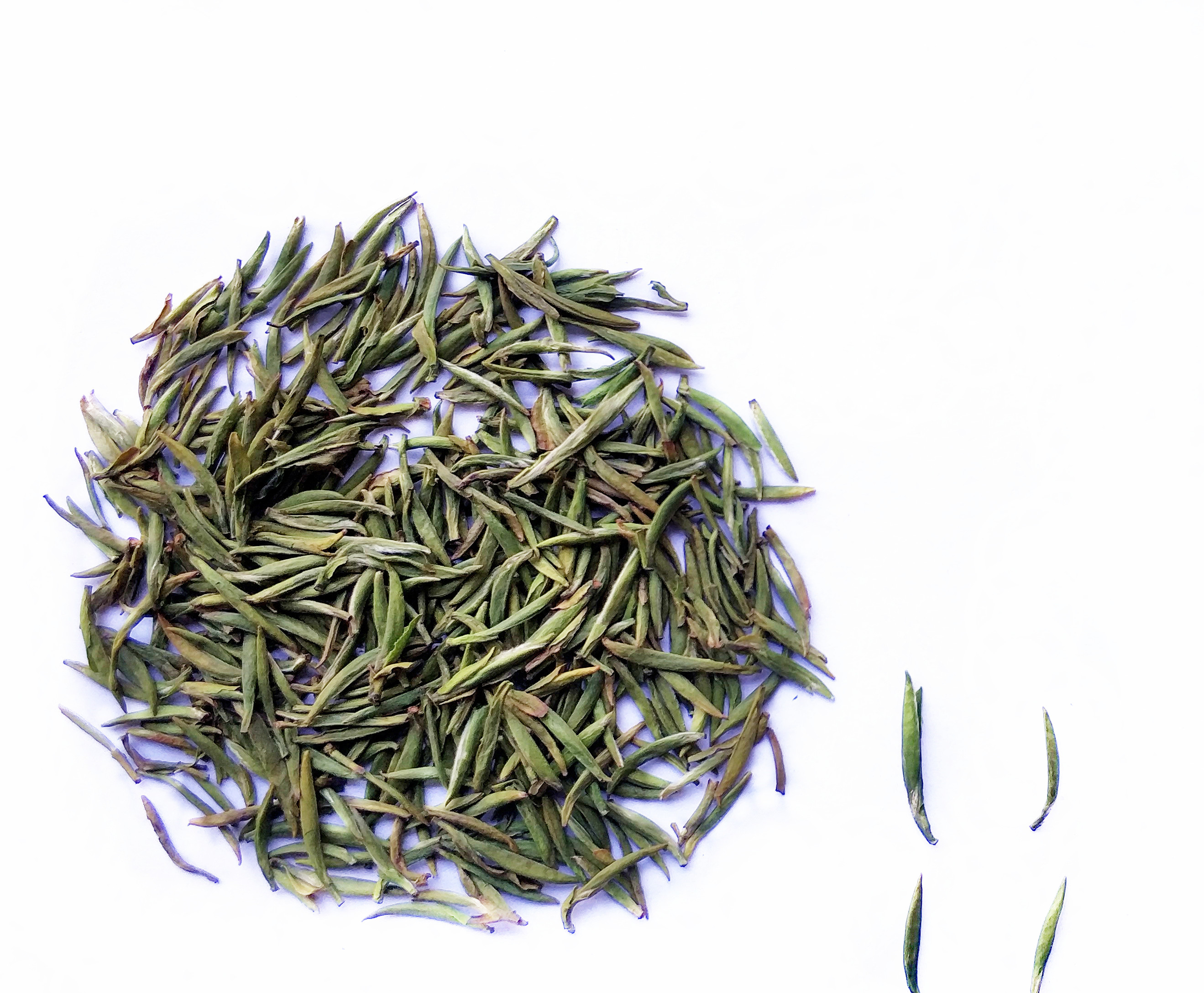 |
| --- | --- | --- | --- |
| **Spreading** | **Fixing** | **Shaping** | **Drying** |
| **Figure S1. BGT processing flow chart** | | | |

| **Ya** | 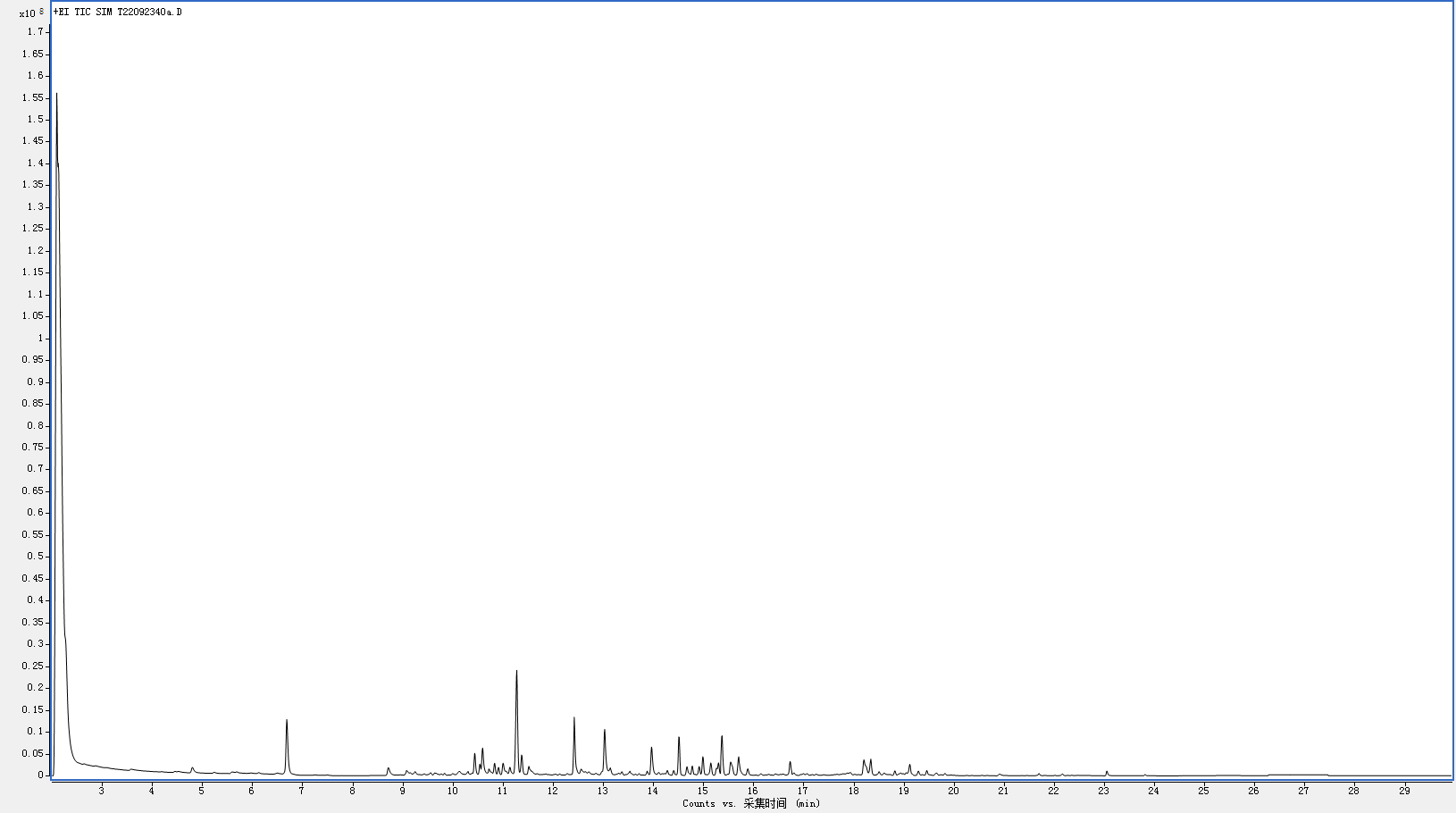 |
| --- | --- |
|  | 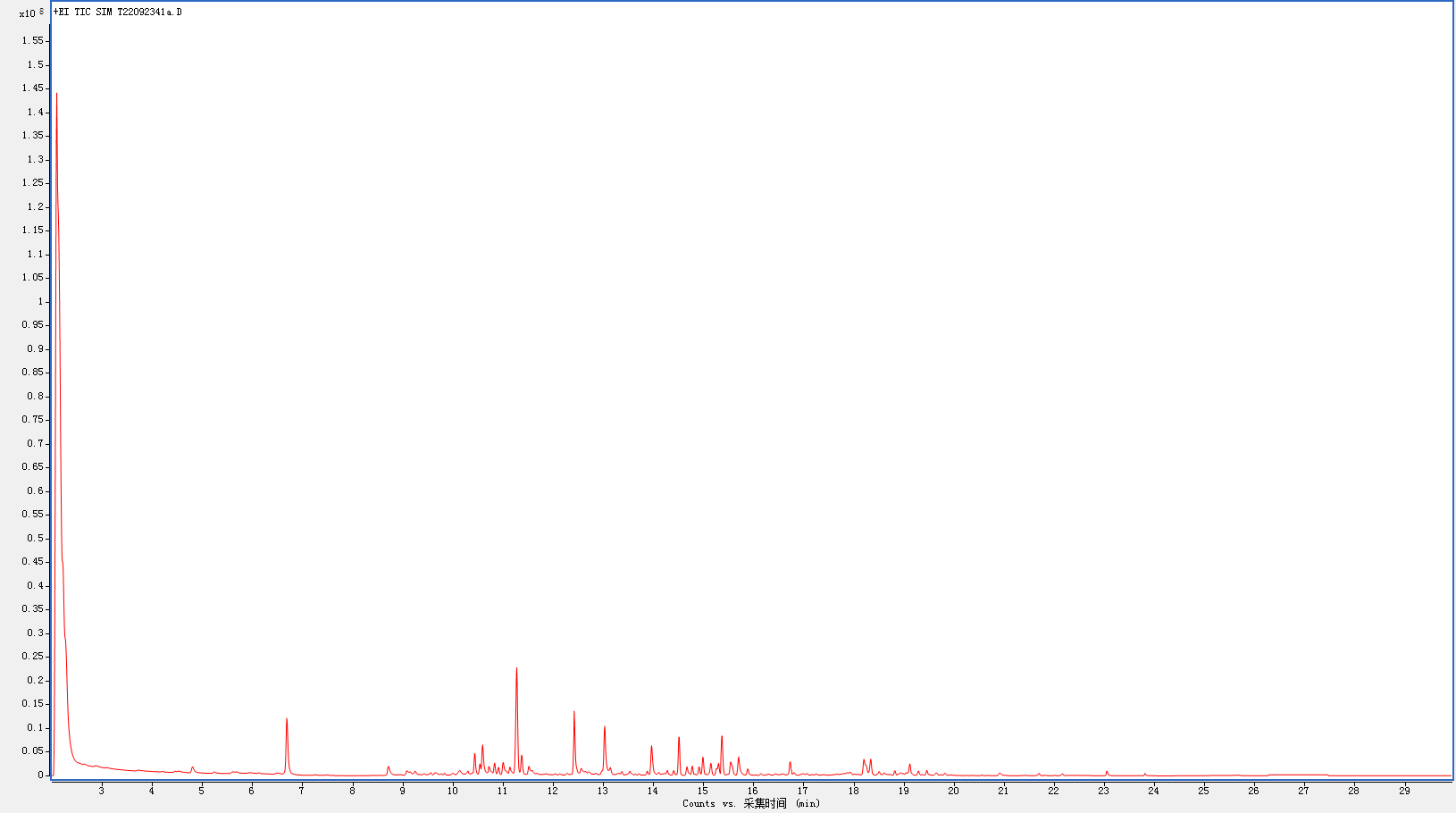 |
|  | 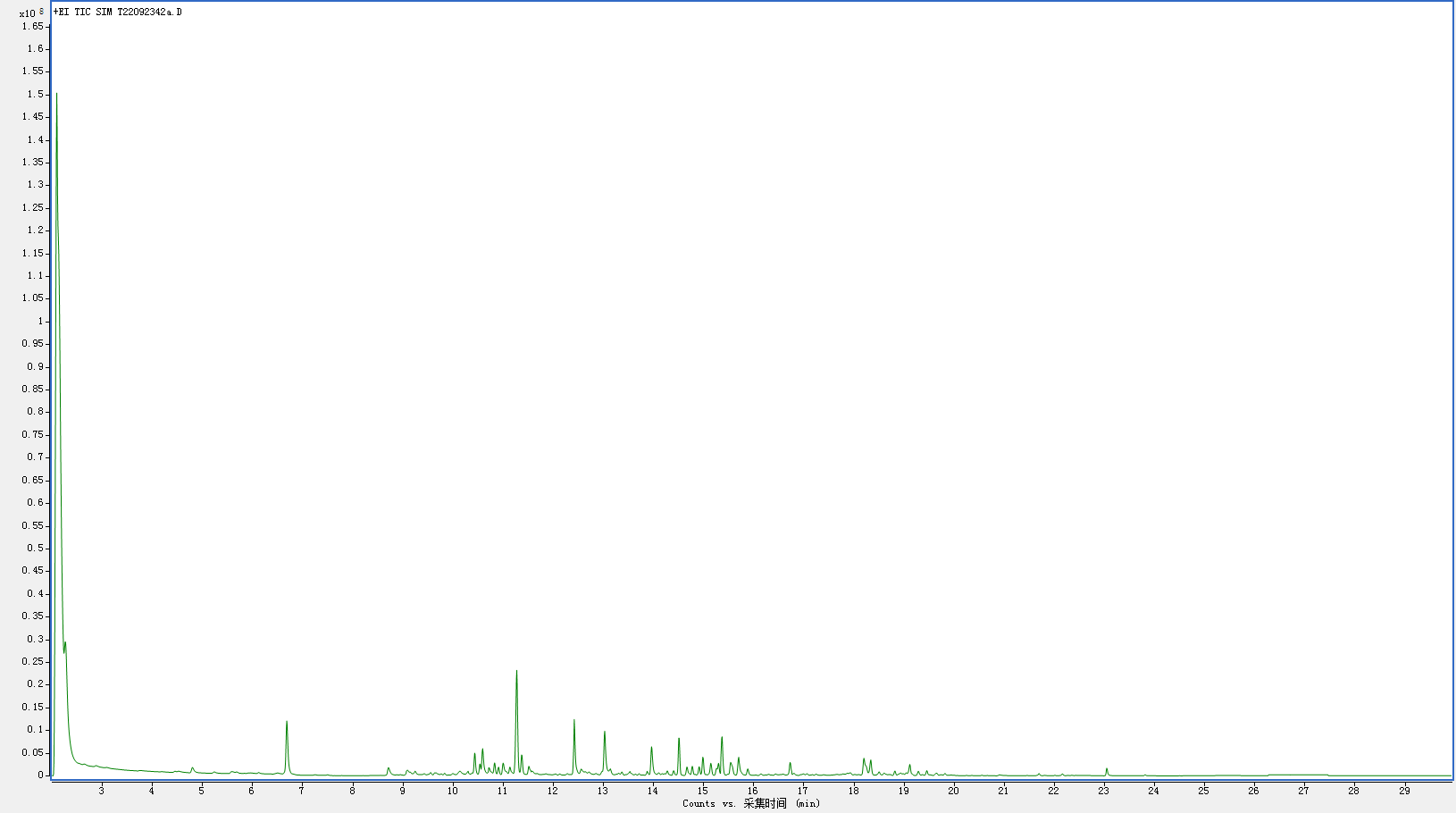 |
| **Yb** | 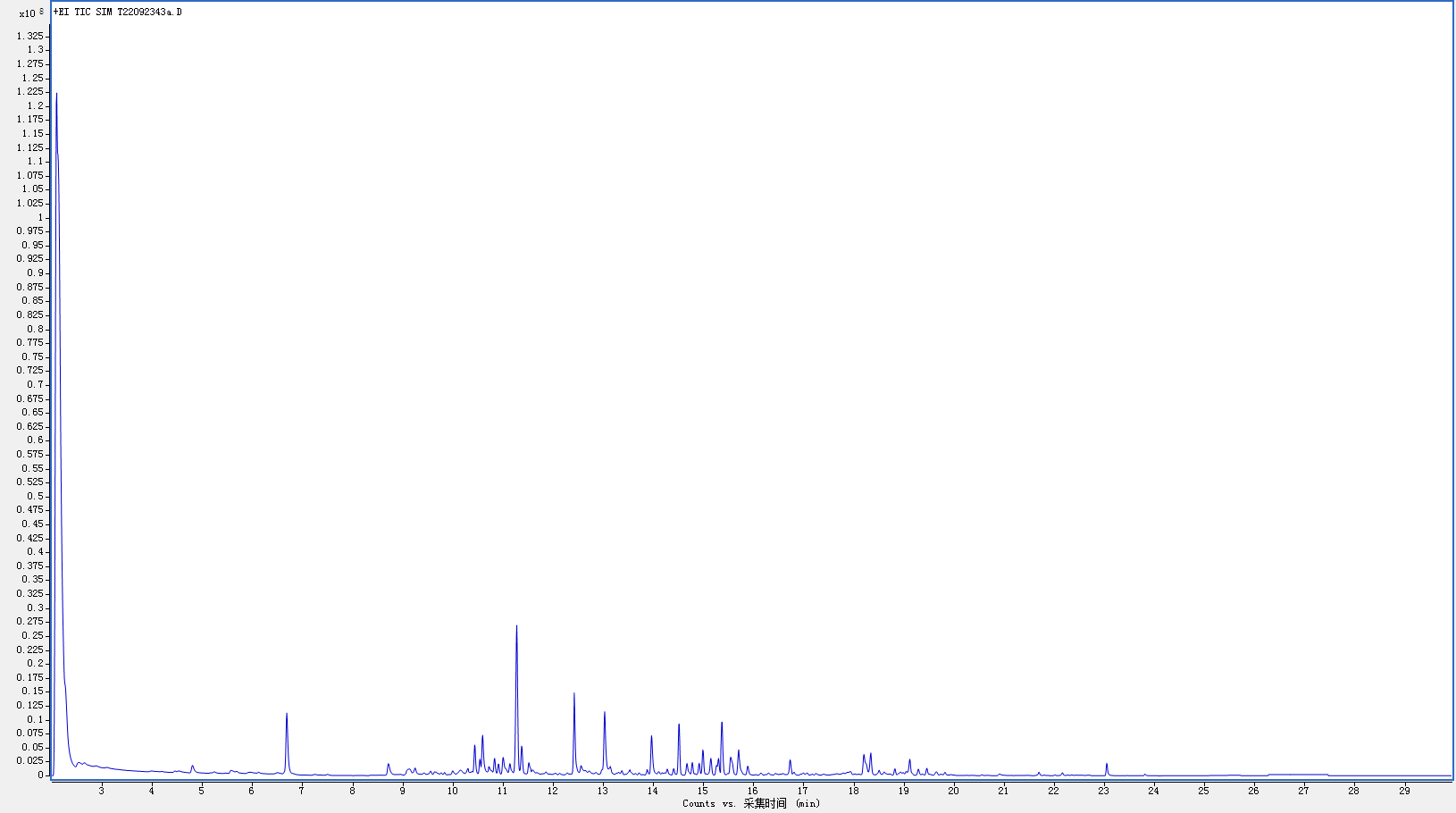 |
|  | 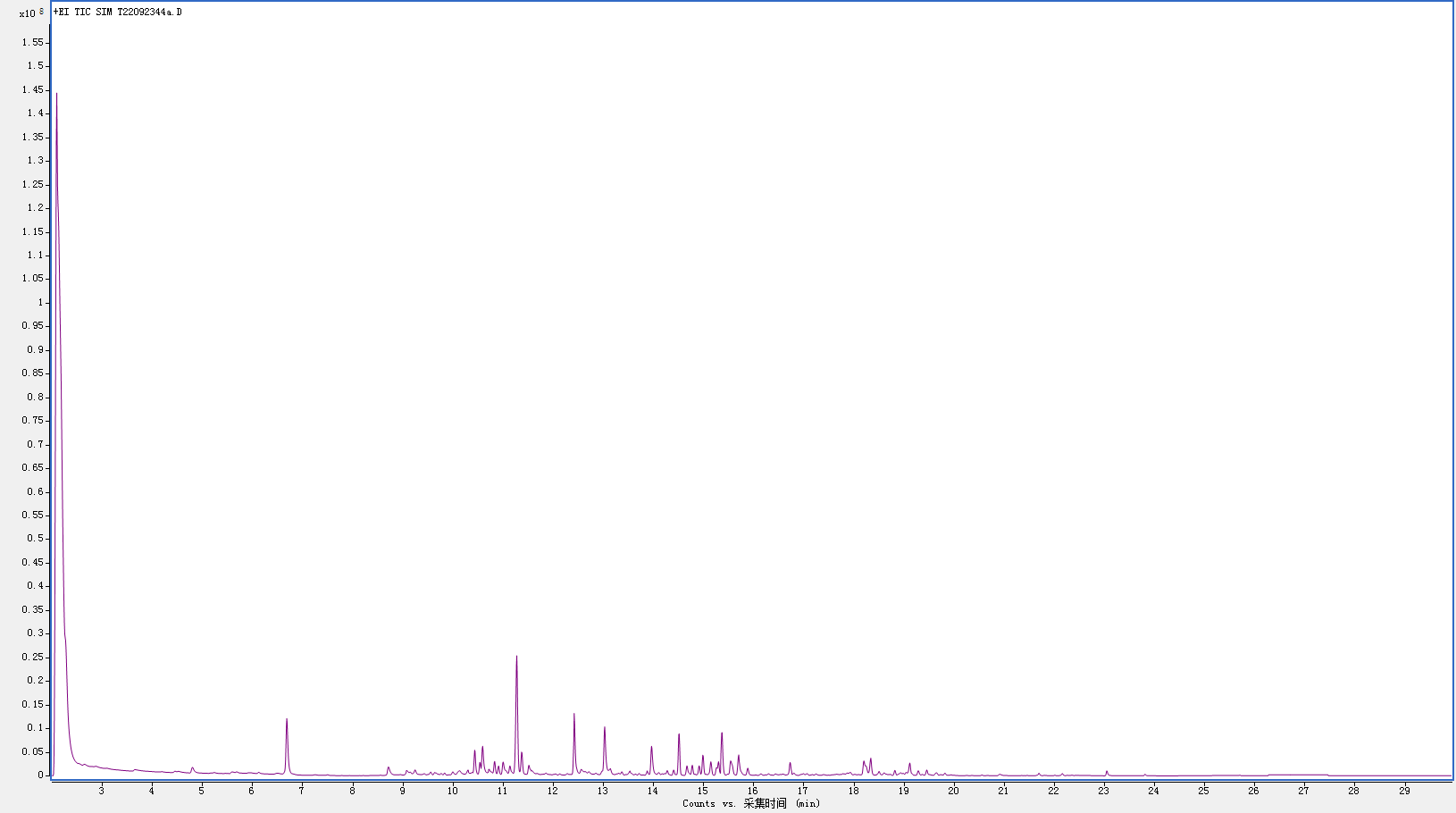 |
|  | 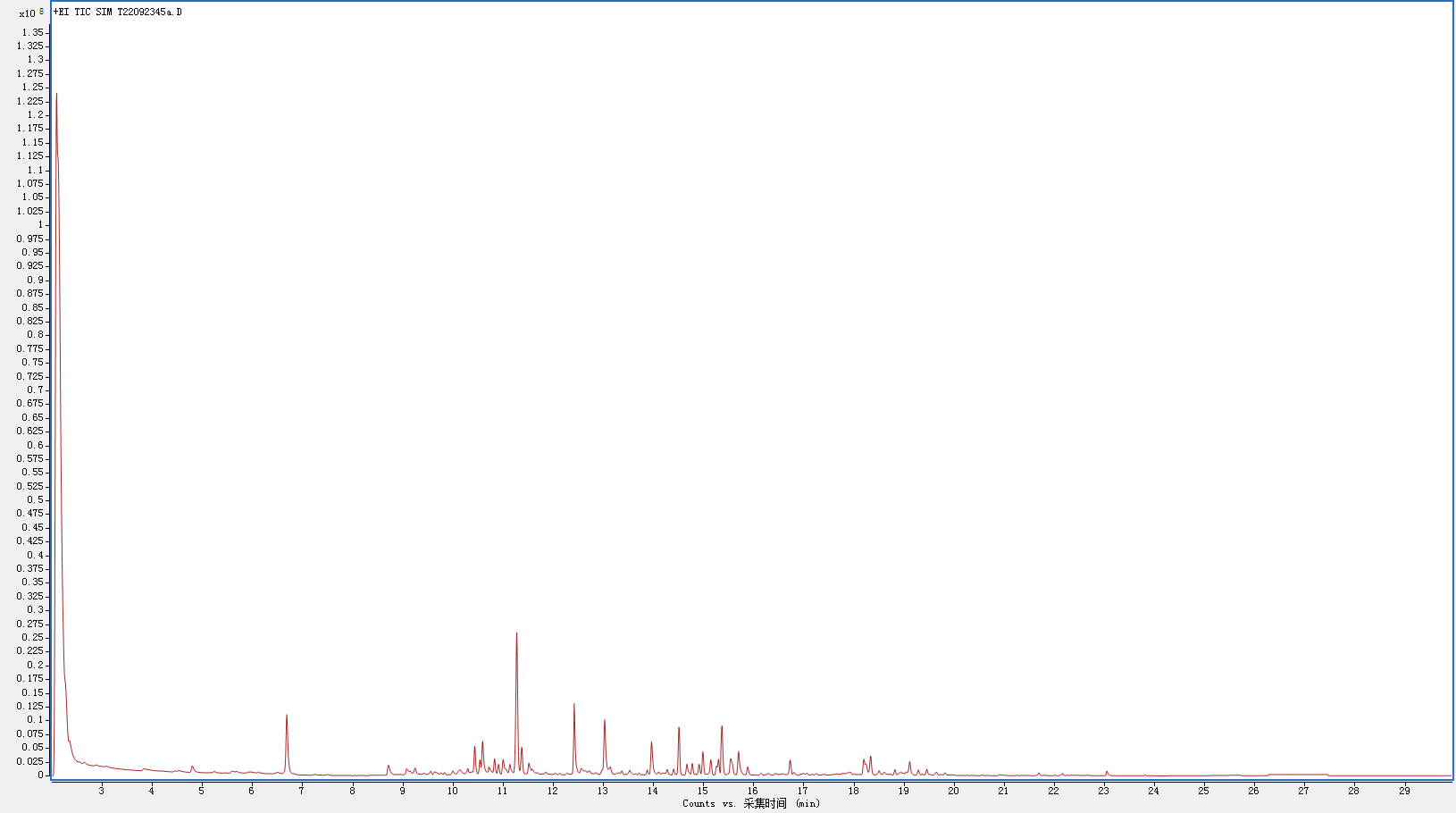 |
| **Yc** | 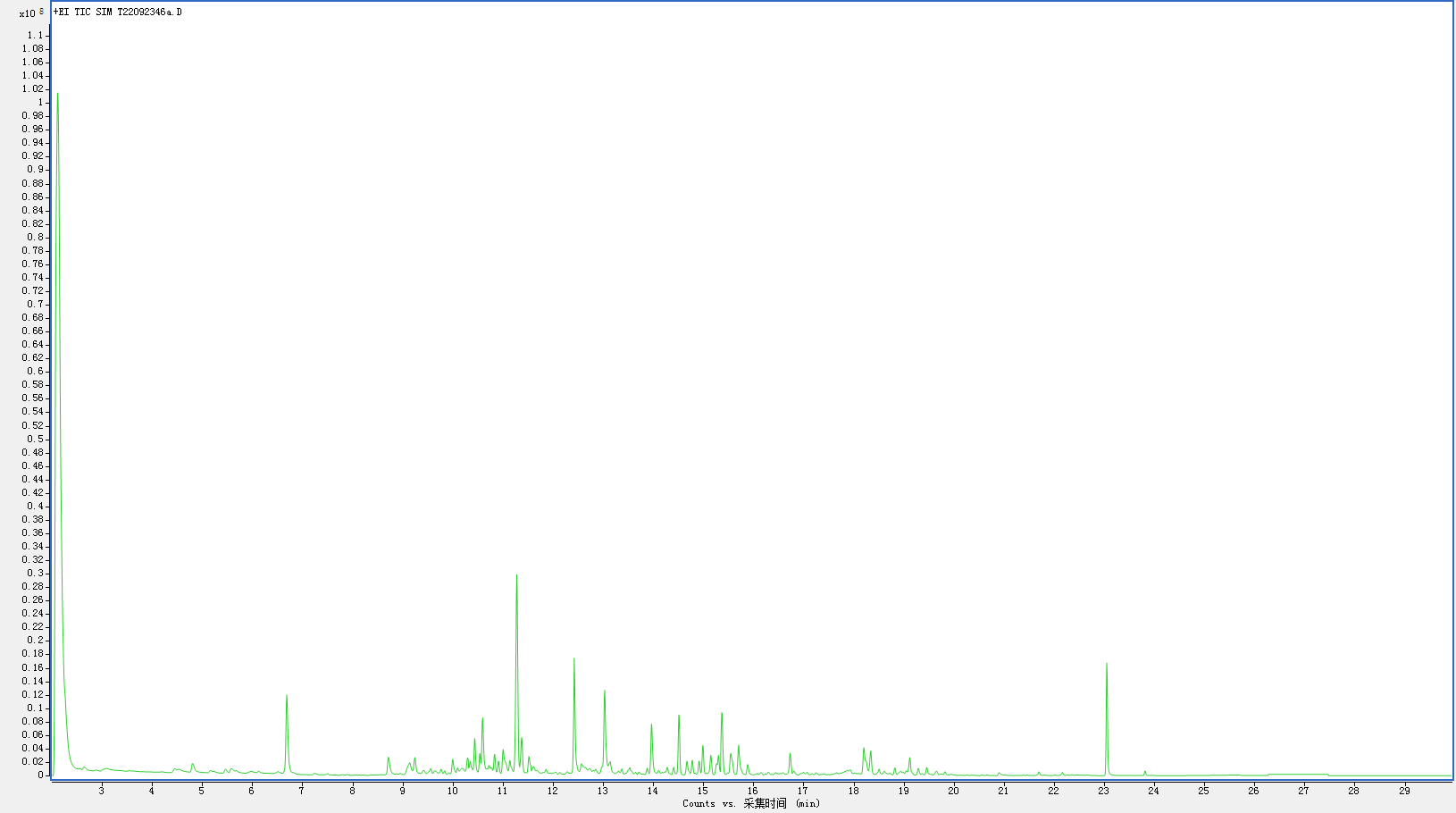 |
|  | 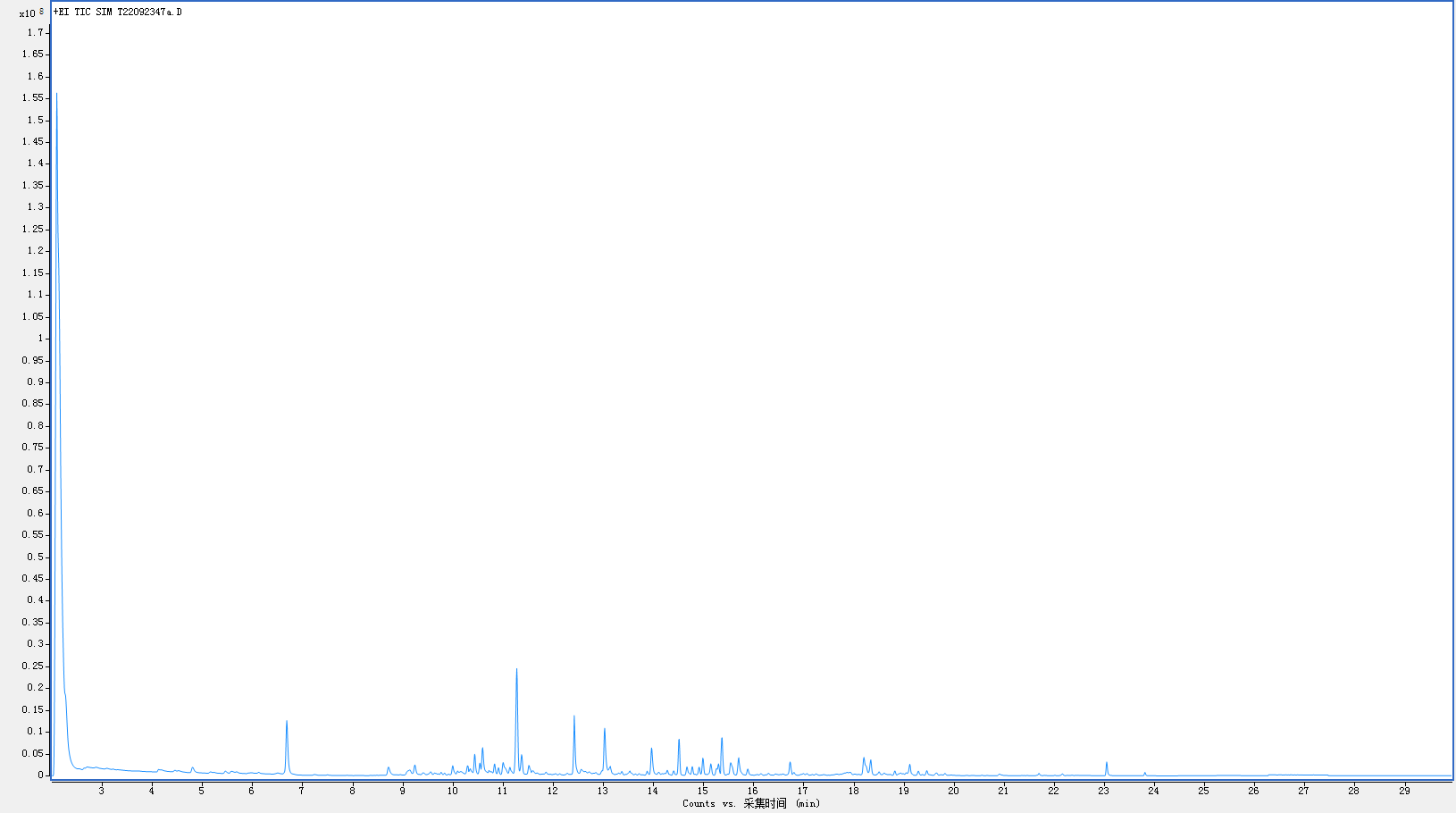 |
|  | 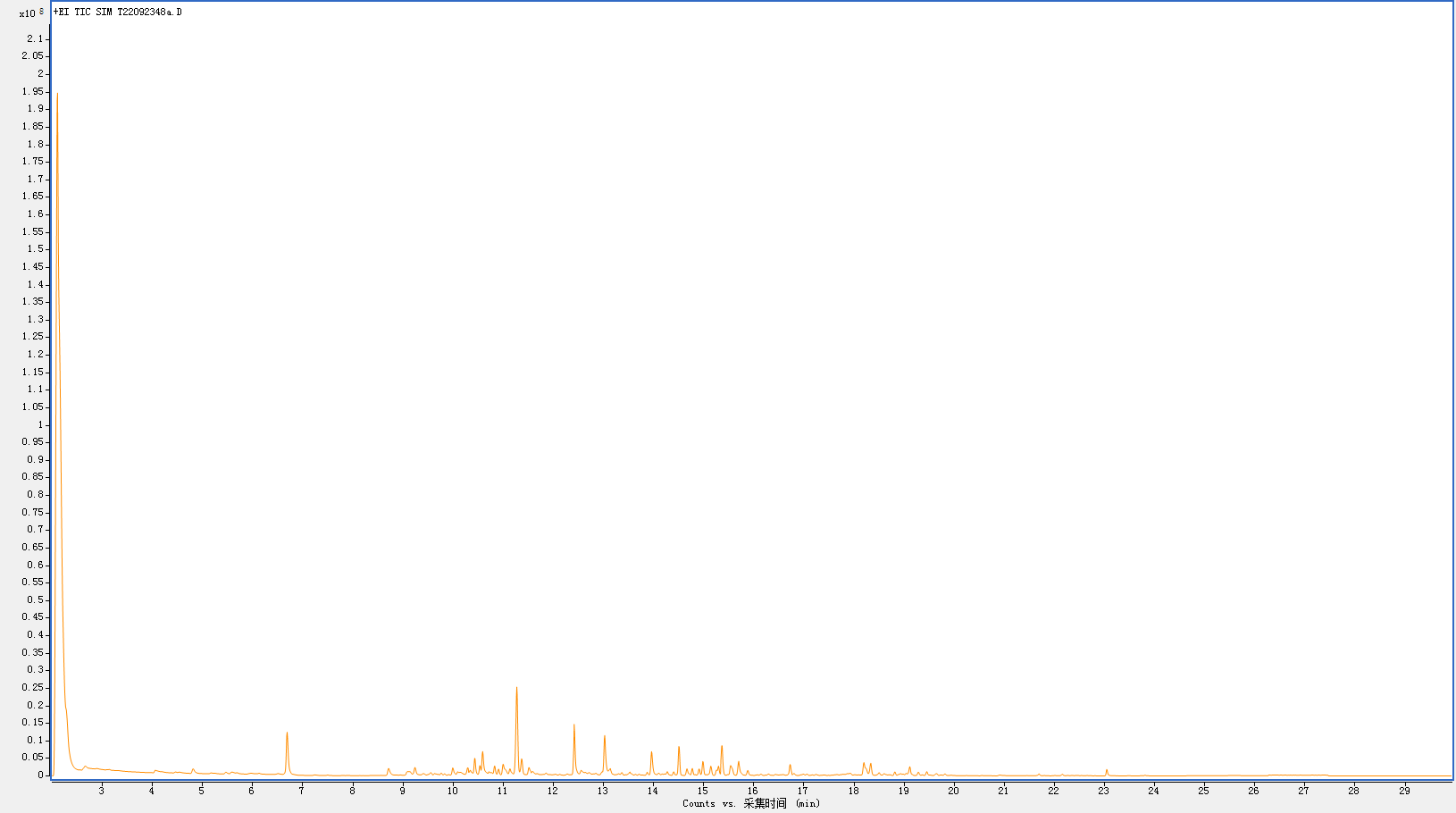 |
| **Yd** | 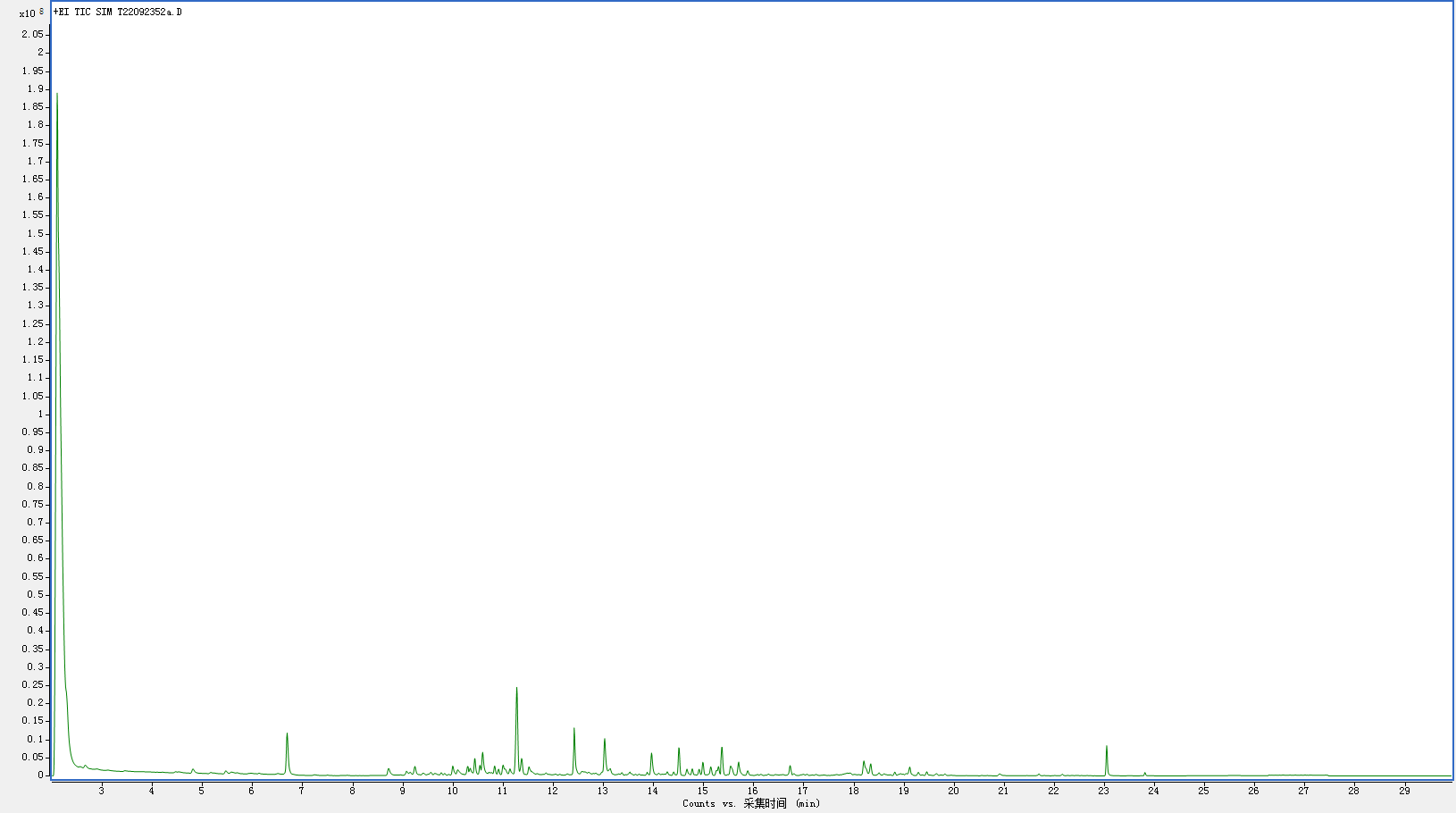 |
|  | 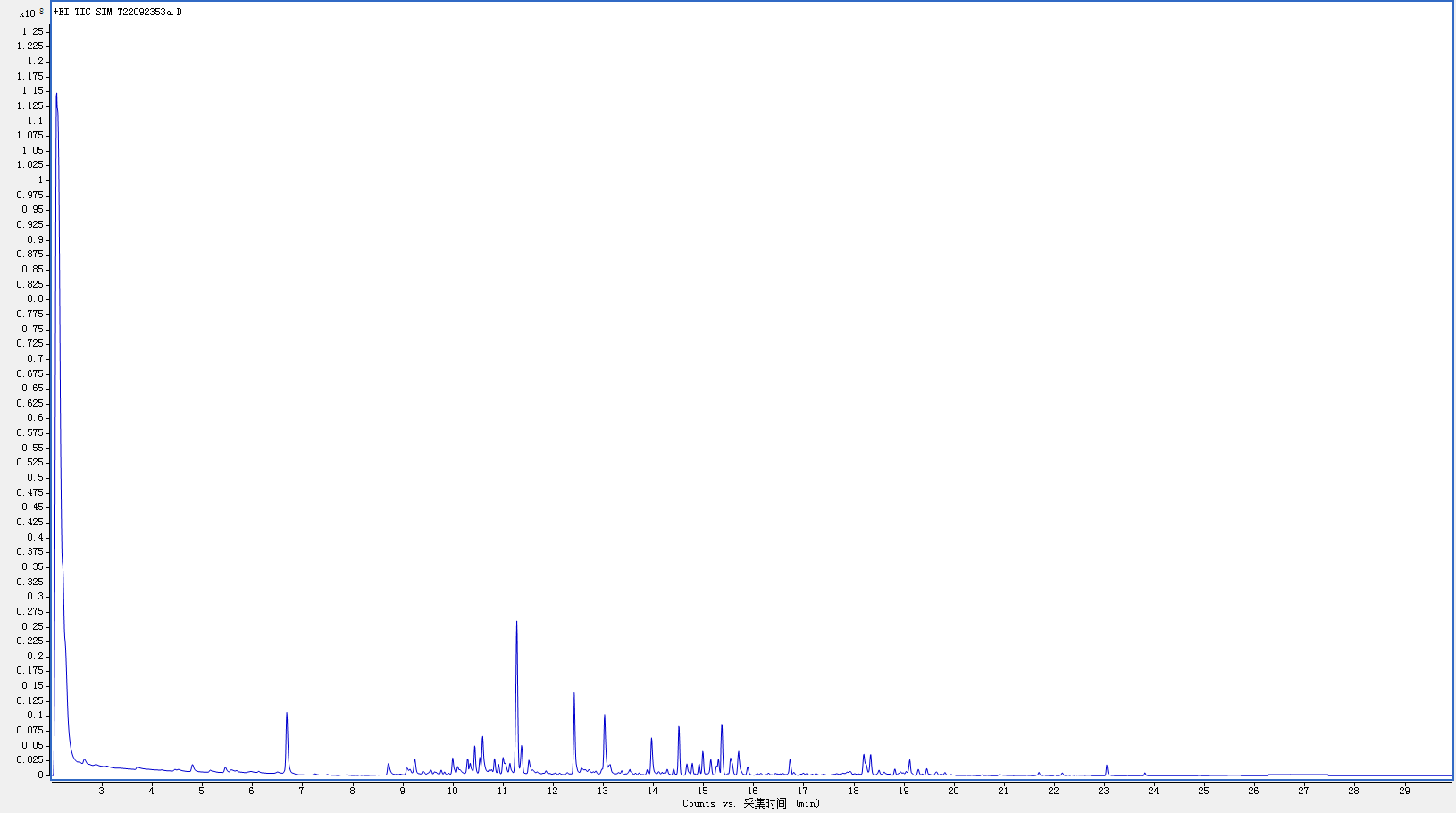 |
|  | 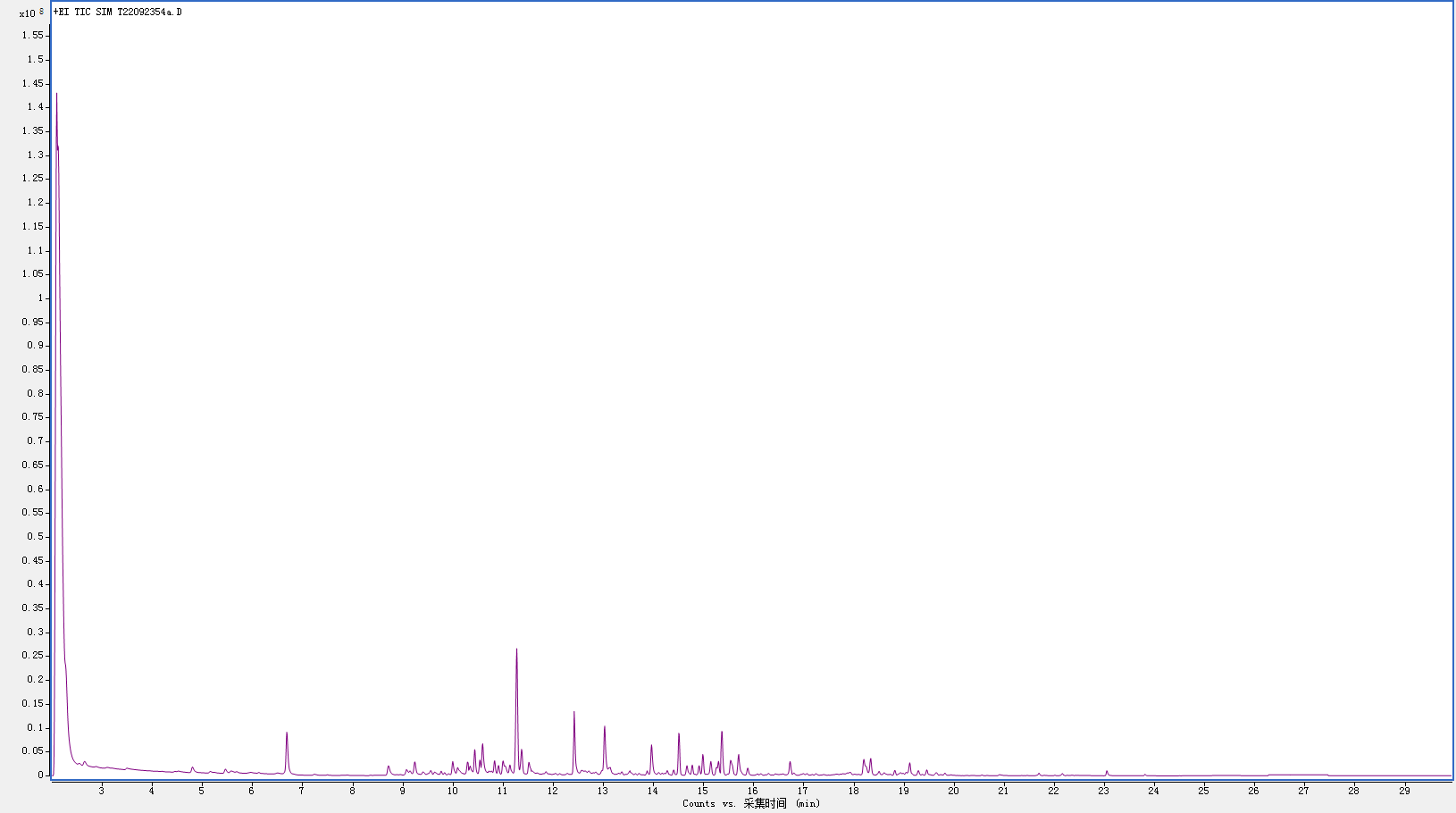 |
|  | **Figure S2. GC-MS spectrum of volatile compounds of bud green tea in different roasting stages**  （Vertical axis = Number X10^8^） |
